# Rheology of the lower mantle: an alternative review

**DOI:** 10.1186/s40645-026-00835-6

**Published:** 2026-07-28

**Authors:** Patrick Cordier, James Van Orman, Philippe Carrez

**Affiliations:** 1 UMR 8207 - UMET - Unité Matériaux Et Transformations, Univ. Lille, CNRS, INRAE, Centrale Lille, 59000 Lille, France; 2 Institut Universitaire de France, 1 Rue Descartes, 75005 Paris, France; 3 Department of Earth, Environmental and Planetary Sciences, Case Western Reserve University, Cleveland, OH 44106 USA

**Keywords:** Rheology, Lower mantle, Mineral physics, Multiscale numerical modelling

## Abstract

Understanding the rheology of the Earth’s lower mantle is essential for modelling mantle convection and its consequences for the geodynamical, thermal and chemical evolution of the planet. However, the extreme pressure–temperature conditions, the long natural timescales, and the complexity of deep-mantle mineralogy make it difficult to constrain mantle viscosity from experiments alone. In this review, we examine an alternative approach based on mineral physics and multiscale numerical modelling, tracing its theoretical foundations and its development over the last decades. We first outline the key physical mechanisms governing plastic deformation in lower-mantle minerals—point defects, dislocations, diffusion, and grain-boundary processes—and the conditions under which they operate. We then summarize the major experimental advances that provide essential constraints for these models. Building on this foundation, we review the evolution of multiscale modelling strategies, from first-principles calculations of defect energetics and dislocation cores to mesoscale dislocation dynamics and polycrystal-scale formulations. Using ferropericlase and bridgmanite as archetypal examples, we show how successive developments have progressively linked atomic-scale mechanisms with macroscopic rheology under deep-mantle conditions. Finally, we discuss current limitations and outstanding challenges, including the scarcity of diffusion data at high pressures, uncertainties in defect chemistry, and the need to integrate multiphase and multimechanism deformation. Together, these elements provide a coherent view of the present capabilities of mineral-physics-based rheology and outline a roadmap for future efforts to improve our understanding of deep-mantle dynamics. 
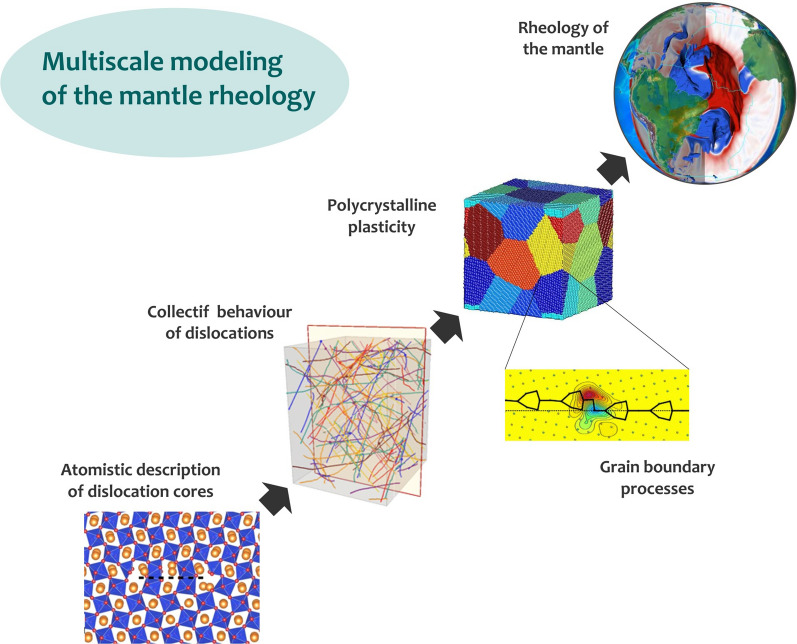

## Introduction

The Earth’s mantle accounts for almost half of its total mass. The lower mantle itself (from 660 to 2890 km depth, from 24 to 136 GPa, and from ∼1900 to ∼4000 K) is the largest region in Earth. It is a solid convective system which transfers internal heat to the surface. Mantle convection encompasses multiple dynamic processes; among them, plate tectonics which reflects the deformation of its cold thermal boundary layer. It also controls the extraction of heat from the core in the hot boundary layer, *i.e.* the core-mantle boundary (CMB). Mantle convection also controls heterogeneous geochemical mixing at depth, as evidenced by the contrasting trace element compositions between mid-ocean ridge and ocean island basalts (Hofmann 1997). Mantle convection is driven by gravity that is acting on internal density differences and which causes a spatially varying internal stress field that relates to deformation and flow via the rheology of mantle materials, *i.e.* their material behavior under mechanical stress. The rheology of the mantle, particularly that of the lower mantle, is therefore paramount for modelling the dynamics of this convective system and, consequently, the long-term thermal and chemical evolution of our planet, defining its present-day actively deforming state as evidenced by the many occurring solid-Earth natural disasters. Rheology is usually expressed in terms of a viscosity (Pa.s^−1^), defining the material resistance against flow, which, as we shall see, must be considered in a broad sense, *i.e.* as either depending linearly or non-linearly on the deforming stress, and often expresses a composite of deformation producing mechanisms. Therefore, the viscosity of a crystalline solid usually depends on several different microscopic mechanisms responsible for plastic flow (creep) active at the high temperatures and pressures of the lower mantle. Important aspects of the deformation mechanisms that govern lower mantle viscosity and other properties of the mantle are still uncertain and the subject of ongoing research and debate recently reviewed by Karato et al. (2025).

Here, we offer an alternative view of lower-mantle rheology in which computational mineral physics ranging from the atomic scale of dislocation cores, through multi-scale modelling to crystals and polycrystals, and benchmarked where possible against laboratory data, is used to constrain the relevant physical properties of the constituent mineral phases. Our aim is to place these developments within their broader scientific context, showing how successive advances have progressively built upon one another. Because the field has evolved rapidly, some contributions have occasionally been cited in a way that obscures their conceptual continuity and progress (Karato et al. 2025). By revisiting the theoretical foundations and the physical principles that structure this framework, we seek to clarify its coherence and limits.

We first outline the geophysical motivations and the main challenges they pose for mineral-physics-based rheology (Sect. 2), before reviewing the theoretical concepts and deformation mechanisms that underpin constitutive laws (Sect. 3.1) and the experimental strategies that constrain them (Sect. 3.2). Section 3.3 then provides a critical assessment of the scaling assumptions traditionally used to extrapolate laboratory data to natural conditions. In contrast, Sect. 3.4 illustrates how an alternative path emerges when rheology is built by transferring information across scales directly from elementary deformation mechanisms. Building on these foundations, Sect. 4 presents an overview of how this framework has been applied to various mantle minerals, highlighting both successes and remaining uncertainties. Finally, Sect. 5 discusses several conceptual and scientific issues that, in our view, remain open and deserve renewed attention.

## What challenges does mantle convection pose for mineral physics?

In this section, we briefly summarize the geophysical constraints that are relevant for mantle rheology. Our goal is not to review mantle convection in general, but to highlight the parameters that mineral physics must ultimately account for, together with the limitations inherent to this approach.

### Pressure and temperature conditions

The pressure in the lower mantle ranges from 24 GPa at 660 km of depth down to 135 GPa at the core-mantle boundary. This wide range of pressures poses a double challenge for mineral physics. As we will briefly discuss below, these conditions remain largely unconquered at an experimental level with regard to conducting well-controlled deformation experiments. Theoretically, it should be noted that these values of confining pressures are of the same order of magnitude as the elastic constants of the considered mineral phases. This suggests that the structure and properties of minerals will be profoundly altered under these pressures. Just as the phases stables at large depths differ from those accessible to us on the Earth’s surface, we can also expect the mechanical properties, which are closely related to the electronic structure, to be significantly impacted by the deep mantle pressure conditions. Therefore, when considering plasticity at large depths, pressure cannot simply be taken as a mechanical loading condition but is rather a state variable that needs to be considered at the atomic scale from a quantum physics perspective.

The profile of average temperature with depth, as well as its lateral variations, is not as well determined in the lower mantle as it is for pressure. Katsura (2022) proposes an adiabatic temperature profile ranging from 1960 K just below the 660-km discontinuity, to 2587 K at 2800-km depth. It is within the Dʺ layer, the thermal boundary layer encompassing the lowermost ∼ 100 km of the mantle, that uncertainties in temperature and its gradient are greatest, with estimates approaching ∼ 4000 K at the core–mantle boundary (Frost et al. 2022). Consequently, this region is where uncertainties in rheological properties are most pronounced; it is also, as discussed below, the part of the mantle for which the physicochemical nature of the constituent mineral phases remains the most poorly constrained.

### Timescales

From a creep perspective, the strain rate is, strictly speaking, the material response to an applied sustained stress. Nevertheless, the order of magnitude of strain rates in the convecting mantle provides an essential framework for analysis. In particular, it highlights the gap between laboratory deformation rates (usually > 10^–6^ s^−1^) and those operating under natural mantle conditions (predicted < 10^–14^ s^−1^).

The kinematics of convection in the Earth’s interior can be estimated from surface movements. Examples include glacial isostatic adjustment (Kaufmann and Lambeck 2002) and geodesy. Plate kinematics and slab sinking flow speeds, together with tomography (*e.g.* van der Meer et al. 2018), can be used to constrain 2D and 3D geodynamic flow models that explicitly predict present-day strain-rate fields (Becker 2006; Alisic et al. 2010; Van der Wiel et al. 2024). While there are variations, all these approaches consistently yield strain rates of the order of 10⁻^15^–10⁻^16^ s⁻^1^ even in the Dʺ layer (Li et al. 2024).

Apart from our recent study (Coulombier et al. 2024), which examined the deformation of olivine glass at room temperature and achieved strain rates close to 10⁻12 s⁻1, experimental access to the extremely low strain rates characteristic of mantle convection remains very limited. The experiment often cited as the slowest, the pitch drop experiment (Edgeworth et al. 1984), corresponds to a strain rate of the order of 10^–8^ s^−1^. More recently, microcreep tests (*e.g.,* Jackson 2019), involving small strain amplitudes (of the order of 10^–5^) over timescales up to 10^4^ s, have pushed this limit further, routinely accessing strain rates as low as ~ 10^–9^ s^−1^. While representing a significant advance, these values still remain several orders of magnitude higher than those of the convective mantle.

The contexts that most closely resemble mantle strain rates are found in the interiors of large ice sheets (Greenland, Antarctic plateau, *e.g.* Dome C, Legresy et al. 2000), where strain rates of 10^–11^−10^–12^ s⁻1 are observed. These environments lend themselves to microstructural analysis, but the behavior of ice cannot be used as an analogue for mantle silicates. It must be acknowledged that we have no direct observations of the microstructure and mechanisms of a silicate sample deforming under high confining pressure, at high temperature and under extremely low strain-rate.

### Which materials?

Petrological models of the lower mantle which are compatible with seismological observables (*e.g.* seismic velocities, density, seismic discontinuities) are constructed for plausible bulk chemistries (*e.g.* pyrolitic/chondritic compositions). These models are created by combining high-P–T experiments and first-principles calculations to map stable phases. This work has led to the identification of the major phases on which efforts are focused to determine the rheological behavior of the lower mantle. These phases are ferropericlase, bridgmanite, davemaoite and, for D", post-perovskite. Research has been conducted on other phases, including high-pressure (HP) polymorphs of silica (*e.g.* stishovite, Texier and Cordier 2006; Kaercher et al. 2015) and dense hydrous phases phases (superhydrous B, Mussi et al. 2013; Phase D, Wu et al. 2024; Phase Egg, Kattemalavadi et al. 2025). However, these studies have been limited in scope and their findings have not been incorporated into global rheological laws. It is clear that we are not close to achieving this latter goal, even for the most significant phases, where the question of the phase that dominates rheology (*e.g.* ferropericlase versus bridgmanite) is frequently prioritized. Even for these key phases, significant simplifications are often required. Bridgmanite, for example, is rarely treated in terms of its full major-element composition, (Mg,Fe,Al)(Si,Fe,Al)O_3_, and is more commonly approximated as (Mg,Fe)SiO_3_ or even as the end-member MgSiO_3_. Even less consideration is given to minor elements (except for hydrogen), redox state and vacancy concentrations. Because experiments at lower mantle pressures are restricted to extremely small sample volumes, it is difficult to produce representative polyphase mineral aggregates. To compensate, experiments require very fine grain sizes, which in turn alter deformation mechanisms and rheological behavior relative to natural lower mantle materials.

## Mineral physics-based approaches to mantle rheology

### Theoretical concepts

Plastic deformation can be described at three complementary levels: (i) the defects that carry deformation, (ii) the elementary processes that govern their motion, and (iii) the macroscopic creep regimes that emerge from their interaction.

#### Fundamental carriers of plastic deformation

##### Point defects

It is well established that point defects can significantly influence the mechanical properties of crystalline materials, either directly or through interactions with other defects such as dislocations. Point defects may occur as vacancies, interstitials (self-interstitials or impurity atoms located between host lattice sites), or substitutional defects (foreign species occupying lattice sites). They are commonly characterized by their formation energies or enthalpies and their corresponding formation volumes.

For intrinsic point defects, the configurational entropy contributes to an equilibrium defect concentration that increases with temperature. In mineral phases, the electric charge associated with point defects must also be considered, leading to the formation of paired defects such as Schottky (pairs of vacancies with opposite charges) or Frenkel defect (a vacancy paired with its corresponding self-interstitial) configurations. For example, in magnesium oxide (MgO), the stable Schottky defect consists of a bound pair of vacancies carrying opposite charges (Yang and Flynn 1994).

The presence of aliovalent impurities—or cations capable of changing oxidation state—can modify the concentration of vacancies, producing what is termed extrinsic defect behavior. A classic example is wüstite (Fe₁₋ₓO), in which iron can change its oxidation state through reactions involving oxygen, thereby altering the vacancy concentration:

1 $$\frac{1}{2}{O}_{2}\left(g\right)+2{\mathrm{F}\mathrm{e}}^{2+}\to {O}^{2-}+{V}_{\mathrm{F}\mathrm{e}}^{{\prime}{\prime}}+2{\mathrm{F}\mathrm{e}}^{3+}$$

${V}_{\mathrm{F}\mathrm{e}}^{{\prime}{\prime}}$ is a vacancy on the ${\mathrm{F}\mathrm{e}}^{2+}$ sublattice with effective charge 2-. Applying the law of mass action to this reaction yields an ${\mathrm{F}\mathrm{e}}^{2+}$ vacancy concentration that depends on the oxygen partial pressure.

##### Dislocations

Dislocations are linear defects characterized by the Burgers vector ***b***, which represents the discontinuity of the displacement field across the line. In the framework of linear elasticity, they are associated with long-range stress and strain fields that decay as 1/r. This slow decay implies that dislocations interact over large distances, leading to collective effects that play a central role in plastic deformation. In contrast to point defects, whose concentration is governed by thermodynamic equilibrium, dislocations are not equilibrium defects. Their density is controlled by the history of deformation through the balance between their generation, motion, and annihilation. As a result, dislocation populations are inherently dynamic and microstructure-dependent. At the same time, the elastic description breaks down in the immediate vicinity of the dislocation line, where the strain becomes very large. In this core region, the continuum approximation is no longer valid and the structure of the dislocation must be described at the atomic scale. A complete description of dislocations therefore requires a multiscale approach, combining continuum elasticity for long-range interactions with atomistic models for the core structure.

##### Grain boundaries

Grain boundaries (as well as phase boundaries) can influence rheology at different levels, becoming more important as grain size decreases. At low temperatures, grain boundaries essentially act as obstacles to intracrystalline plasticity, leading to Hall–Petch law^1^. However, at high temperatures, they can actively contribute to plastic deformation by acting as sources or sinks of vacancies, for example. The effect is then reversed, leading to a lower yield stress.

Low-angle grain boundaries can be described with dislocations. High-angle grain boundaries are typically described in terms of structural units (Priester 2013), but their mechanical properties may be influenced by their defect content, which may include dislocations (Priester 2013), disclinations (Cordier et al. 2014), or disconnections (Han et al. 2018; Hirth et al. 2020).

#### Elementary deformation processes

##### Atomic transport by diffusion

Atomic transport by diffusion arises from the thermally activated motion of point defects, most commonly vacancies, which enable atoms to migrate through the crystal lattice. At finite temperature, an atom can exchange positions with a neighboring vacancy, resulting in transport of atoms opposite to the direction of vacancy motion. Although interstitial atoms or ions can in principle contribute to matter transport, vacancy migration is usually the dominant mechanism. This is why vacancy concentration, and the formation of Schottky defects, is of central importance. Because interstitial defects typically have high formation energies, the concentration of Frenkel defects is often negligible compared to that of Schottky defects.

If a gradient in vacancy concentration exists within the crystal, this leads to a vacancy flux proportional to that gradient, following Fick’s law:

2 $${\boldsymbol{J}}=-{D}_{\mathrm{s}\mathrm{d}} \mathbf{g}\mathbf{r}\mathbf{a}\mathbf{d}C$$

where ${D}_{sd}={D}_{O}{\mathrm{exp}}\left(-\frac{{\Delta H}_{\mathrm{s}\mathrm{d}}}{kT}\right)$ is the self-diffusion coefficient. In this expression, ${D}_{O}$ incorporates both the attempt frequency and the entropy term associated with the migration jump. In the intrinsic regime, where vacancies are thermally generated and their concentration is governed by equilibrium defect formation, the activation enthalpy for self-diffusion (${\Delta H}_{\mathrm{s}\mathrm{d}}$) is the sum of the vacancy formation and migration enthalpies. In the extrinsic regime, by contrast, vacancy concentrations are fixed by external conditions such as impurity content or oxygen partial pressure, and ${\Delta H}_{\mathrm{s}\mathrm{d}}$ contains only the migration enthalpy, as the vacancy concentration is fixed by extrinsic factors rather than by thermodynamic equilibrium.

In real crystals, diffusion does not occur in a homogeneous medium but is strongly influenced by the presence of defects and interfaces. Migration barriers are highly sensitive to the local atomic environment (see, for example, Mahmoud et al. 2021), and diffusion is therefore often significantly enhanced in structurally perturbed regions such as dislocation cores and grain boundaries. Diffusion along dislocation cores, commonly referred to as pipe diffusion, has been observed experimentally and numerically in a wide range of systems, including metals and silicates (Legros et al. 2008; Gaboriaud 2009; Yund et al. 1981; Yurimoto et al. 1992; Sakaguchi et al. 1992a,b; Zhang et al. 2010; Landeiro dos Reis et al. 2022). Because migration barriers are reduced in the vicinity of dislocations, pipe diffusion becomes particularly important at low temperatures, where bulk diffusion is sluggish.

Similarly, grain boundaries provide preferential pathways for diffusion (McKenzie et al. 1971; Farver et al. 1994; Harris et al. 1997; Polednia et al. 2020; Riet et al. 2021). This contribution is often expressed through an effective parameter ${D}_{\mathrm{g}\mathrm{b}}\delta$ where δ represents an effective grain boundary width. However, δ should be regarded as a phenomenological parameter rather than a true structural thickness. In ionic solids in particular, enhanced diffusion does not necessarily coincide with the structurally disturbed region observed by transmission electron microscopy (TEM), but may instead occur within adjacent space-charge zones, where point defects responsible for enhanced diffusion can accumulate (Marquardt et al. 2011; Parras et al. 2021).

These different diffusion pathways illustrate that atomic transport is intrinsically coupled to the defect structure of the material, and cannot be considered independently of dislocations and interfaces. Diffusion thus provides the fundamental mechanism by which matter is redistributed in response to stress, forming the basis of diffusional creep processes discussed below.

##### Strain transport by dislocations

Strain transport by dislocations results from the motion of line defects under stress, through a combination of glide and climb processes.

**Fig. 1:**
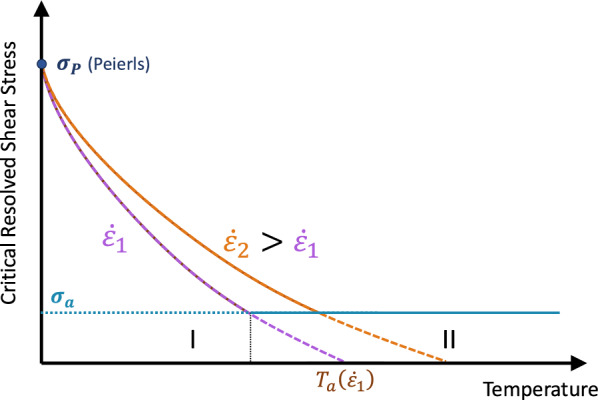
Thermal activation of dislocation glide. Below a critical temperature (the so-called athermal temperature *T*_*a*_) which depends on the strain-rate, dislocation glide assisted by the kink-pair mechanism is thermally activated. In practice, before reaching temperature *T*_*a*_, the stress stabilizes on a plateau (${\sigma}_{a}$) resulting from interactions at long (elastic) or short (intersections, junctions) distances between dislocations

*Glide.* Under the influence of shear stress, the dislocation can move conservatively in a plane (the glide plane of unit plane normal ***n***) defined by the Burgers line and vector. This displacement produces a shear proportional to ${\boldsymbol{b}}\otimes {\boldsymbol{n}}$ (Kocks et al. 1975)**.** In order to understand the dynamics of dislocations, it is first necessary to understand their velocity. This velocity is never the theoretical velocity of shear waves, at least under the conditions of a convective mantle. In practice, the velocity of dislocations is always extrinsic and is determined by their interaction with ‘obstacles’. Therefore, dislocations only become mobile if they manage to overcome an average resistance to glide. This is important to know in order to determine the yield strength. Peierls demonstrated that the crystal lattice was the source of periodic lattice friction. Understanding Peierls friction for each slip system (slip direction and slip plane) is important for appreciating plastic anisotropy. The Peierls friction stress ${\sigma}_{P}$ is often assimilated to the critical resolved shear stress (CRSS) at absolute zero temperature. At finite temperatures, overcoming Peierls barriers is facilitated by thermal agitation using the kink-pair mechanism. As the temperature increases, the kink-pair mechanism evolves into a viscous drag regime. In this regime, the nucleation or motion of kinks along the line is no longer the limiting process, but rather it is the slowing down of dislocation motion due to phonon interactions. In this high-temperature regime, dislocation lines have no preferential characteristics and their velocity is constrained by a viscous drag attributed to interactions between dislocations and phonons. This velocity is then proportional to the resolved shear stress (τ):

3 $$v\left(\tau ,T\right)=\frac{b\tau }{B\left(T\right)}$$

where $B\left(T\right)$ is a viscous drag coefficient generally of the order of 10^–5^ Pa.s in ionic crystals. Interactions between moving dislocations and ‘obstacles’ often lead to the definition of a characteristic temperature, known as the athermal temperature (*T*_*a*_), at which point flow becomes essentially athermal. The athermal temperature can arise in the kink-pair regime of motion, but is more commonly encountered in drag- or interaction-controlled regimes. By ‘athermal’, one means that the motion of the dislocation is controlled by long-range interactions, such as those between dislocations. Therefore, the stress felt by the dislocation between two obstacles is large enough to ensure a ‘free’ glide regime. However, the effective average velocity is more dependent on waiting time at obstacles than on free glide velocity. Figure 1 schematically illustrates the evolution of resistance to specific obstacles as a function of temperature. It is frequently associated with the evolution of CRSS, *i.e.* the microscopic yield strength as a function of temperature. This method is not appropriate for describing a steady-state creep regime. Instead, a different approach is required (see below).

*Climb.* By absorbing or emitting point defects, a dislocation with an edge component can also move in a plane perpendicular to the Burgers vector, the climb plane, producing a normal strain along the direction of the Burgers vector proportional to ${\boldsymbol{b}}\otimes {\boldsymbol{b}}$. There is no rotation component in the latter case. Being controlled by the diffusion of point defects, climb is therefore strongly temperature-dependent. It enables dislocations to bypass obstacles and plays a central role in high-temperature deformation regimes. More fundamentally, climb provides the physical basis for recovery processes, allowing dislocations to annihilate, rearrange, or change their configuration. Through these mechanisms, climb contributes to the evolution of dislocation density and microstructure, and therefore to the establishment of steady-state creep regimes discussed below.

##### Grain-boundary–mediated deformation

In addition to atomic transport by diffusion and strain transport by dislocations, grain boundaries provide a third class of deformation processes. These interfaces can accommodate deformation by moving along their plane (sliding), normal to it (migration) or in an inclined direction by a process called shear-coupled migration. In such processes, deformation is mediated by the motion and structural rearrangement of interfaces, often involving a coupling between atomic transport and dislocation activity. As a result, grain-boundary–mediated deformation occupies an intermediate position between diffusion-controlled and dislocation-mediated processes. These processes highlight the key role of interfaces as active carriers of deformation, rather than passive structural features.

#### From processes to creep regimes

The elementary processes described above form the basis from which macroscopic creep regimes emerge, depending on which mechanisms dominate under given conditions. Creep is a general term describing time-dependent irreversible deformation under sustained stress. Its precise meaning varies across communities: in materials science it usually refers to thermally activated plastic flow at high temperature, while in geophysics it may denote any long-term viscous or viscoplastic deformation, sometimes encompassing mechanisms that are physically distinct. In both communities, particular emphasis is often placed on steady-state (secondary) creep, in which the strain rate becomes time-independent after an initial transient. The conditions under which such a regime is attained and its physical interpretation may, however, differ. Care is therefore required, as identical terminology can refer to different underlying processes in different fields. In the following, we focus primarily on steady-state creep, which provides the most robust framework for defining constitutive laws. To provide a coherent framework for the discussion that follows, Table 1 summarizes the main creep regimes, their controlling mechanisms, and their associated constitutive laws. It should be emphasized that these categories do not represent strictly separate domains, but rather idealized end-members of a continuum of deformation behaviors arising from the coupling between diffusion, dislocation motion, and microstructural evolution.

**Table 1:** Overview of the principal creep regimes discussed in this review

Creep regimes	Strain-producing mechanism	Rate-limiting process	Governing constitutive form	Stress exponent (*n*)	Grain size sensitivity (d)	Phenomenological	CPO
Glide-controlled creep	Dislocation glide over lattice friction	Kink-pair nucleation/glide mobility	$\dot{\varepsilon }={\dot{\varepsilon }}_{0}\mathrm{exp}\left[-\frac{\Delta {H}_{0}}{kT}{\left(1-{\left(\sigma /{\sigma}_{P}\right)}^{p}\right)}^{q}\right]$	high, variable	–	No (if derived)	Yes
Recovery-controlled creep	Dislocation glide	Dynamic recovery (often climb-controlled)	$\dot{\varepsilon }={A}_{\mathrm{P}\mathrm{L}}{\sigma }^{n}\mathrm{exp}\left(-\frac{Q}{RT}\right)$	∼3–5	–	Partly	Yes
Nabarro–Herring creep	Mass transfer–Stress-driven lattice diffusion between grain boundaries	Bulk diffusion of slowest species; GBs acting as sources/sinks	$\dot{\varepsilon }={A}_{\mathrm{N}\mathrm{H}}\frac{{D}_{\mathrm{s}\mathrm{d}}\Omega }{kT}\frac{\sigma }{{d}^{2}}$	1	2	No	No
Coble creep	Mass transfer–Grain-boundary diffusion	Grain-boundary diffusion	$\dot{\varepsilon }={A}_{C}\frac{{D}_{\mathrm{g}\mathrm{b}}\delta\Omega }{kT}\frac{\sigma }{{d}^{3}}$	1	3	Partly	No
Pure climb creep	Dislocation climb	Bulk diffusion of slowest species between dislocations acting as vacancy sources/sinks	$\dot{\varepsilon }=\frac{{D}_{\mathrm{s}\mathrm{d}}\mathrm{b}}{\pi kT}\frac{{\sigma }^{3}}{{\mu }^{2}}/\mathrm{ln}\left(\frac{4\mu }{\pi \sigma }\right)$	3	–	No	No

For each regime, the table summarizes the dominant strain-producing mechanism, the rate-limiting process controlling deformation kinetics, the general constitutive form, and the expected microstructural or textural signatures

##### Glide-controlled creep

Before introducing steady-state creep regimes, it is useful to distinguish a class of deformation regimes in which dislocation glide alone controls the strain rate. In such cases, lattice friction dominates over other interactions, leading to deformation modes often referred to (somewhat loosely) as “Peierls” or “low-temperature” creep, or even “plasticity” regimes. Although these regimes can sometimes be approximated by Norton-type laws over a limited range, they are characterized by very high apparent stress exponents (typically *n* > 10) and a strong sensitivity to stress and temperature, reflecting an underlying quasi-exponential dependence. This behavior is more appropriately described using thermally activated models based on kink-pair nucleation (Kocks et al. 1975; Goetze 1978):

4 $$\dot{\varepsilon }={\dot{\varepsilon }}_{0}\mathrm{exp}\left[-\frac{\Delta {H}_{0}}{kT}{\left(1-{\left(\sigma /{\sigma}_{P}\right)}^{p}\right)}^{q}\right]$$

where $\Delta {H}_{0}$ is the critical activation enthalpy at zero stress, *p* and *q* are adjustable parameters, and ${\sigma}_{P}$ is the Peierls stress. If the glide velocity law of dislocations (${v}_{g}$) is known, this mechanism can be modelled based on Orowan’s law, which is a transport equation:

5 $$\dot{\varepsilon }=\rho b{v}_{g}$$

where ρ is the dislocation density, *b* is the modulus of the Burgers vector and ${v}_{g}$ is the average velocity of gliding dislocations if it is defined. These regimes do not generally correspond to steady-state creep in the strict sense, as they do not involve a well-defined balance between hardening and recovery, and are therefore treated separately from the creep regimes discussed below.

##### Power law creep

Most high-temperature deformation regimes, by contrast, fall within the framework of steady-state creep described by power-law relationships. In this case, the strain rate is commonly expressed using a Norton (1929)-type equation:

6 $$\dot{\varepsilon }={A}_{\mathrm{P}\mathrm{L}}{\sigma }^{n}\mathrm{exp}\left(-\frac{Q}{RT}\right)={\dot{\varepsilon }}_{0}{\left(\frac{\sigma }{\mu }\right)}^{n}\mathrm{exp}\left(-\frac{Q}{RT}\right)$$

where *n* is the stress exponent (typically *n* ∼ 3–5), and *Q* an activation energy. It should be noted that this phenomenological formulation (later taken up by other authors, including Bird et al. 1969) predates the modern understanding of dislocations and does not, in itself, identify the underlying mechanisms. In the second expression, the single empirical parameter ${A}_{PL}$ has been split up into two, which have the dimensions of the variables under discussion. In practice, similar values of *n* and *Q* may arise from different physical processes, so that these parameters are not always fully discriminating. The key factor controlling the creep rate is the effective mobility of dislocations, which is generally extrinsic and governed by the obstacles they must overcome. The following sections therefore adopt a mechanism-based classification, emphasizing the physical processes controlling deformation rather than purely phenomenological distinctions.

##### Recovery-controlled creep

A first major class of mechanisms corresponds to recovery-controlled creep. In this case, dislocation glide is not rate-limiting; instead, deformation is controlled by the ability of the system to reorganize its microstructure through recovery processes. The steady-state creep rate can be expressed formally using the Bailey-Orowan equation $\dot{\varepsilon }=r/h$, where *r* and *h* are the recovery and strain hardening rates respectively. We can enrich Orowan’s law by taking these two processes into account and outlining a law of microstructure evolution described by dislocation density as a state variable. Mecking and Kocks (1981) showed that, since the evolution of dislocation density results from competition between the recovery and storage rates of dislocations, it could be described by an equation of the form

7 $$\frac{d\rho }{d\varepsilon }={k}_{1}\sqrt{\rho }-{k}_{2}\rho$$

where the first term with ${k}_{1}$ represents dislocation storage (*e.g.*, forest^2^ interactions, dipole formation) and the second term with ${k}_{2}$ represents dynamic recovery. Although several processes may contribute to recovery, climb-controlled mechanisms are most often dominant. In the classical model proposed by Weertman (1955), dislocations glide until blocked by interactions forming dipoles, which are subsequently annihilated by climb. This framework naturally leads to stress exponents in the range n∼3-5 (Weertman 1968).

##### Diffusional creep

A second class of mechanisms corresponds to diffusional creep, here considered in a broader sense than the classical definition. Rather than restricting this term to grain-boundary–mediated diffusion, we include all deformation processes involving the transport of point defects interacting with microstructural features such as grain boundaries or dislocations.

###### Nabarro-Herring and Coble creep

**Fig. 2:**
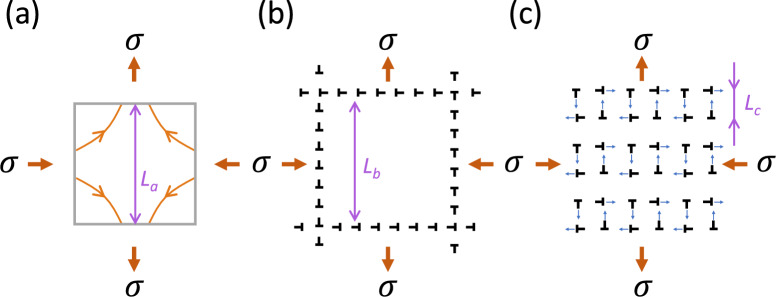
Different mechanisms of diffusional creep. **a** Nabarro-Herring creep **b** Subgrain undergoing Nabarro-Herring creep **c** Pure climb (Nabarro) creep. Small arrows indicate climb directions. ${L}_{i}$ corresponds to the characteristic diffusion lengths: grain size (${L}_{a}$), cell size (${L}_{b}$) and dislocation spacing (${L}_{c}\sim 1/\sqrt{\rho }$, where ρ is the dislocation density)

The mobility of point defects enables the transport of matter, which, when directed by applied stress, can produce plastic work. Nabarro (1948) was the first to demonstrate that a differential stress field could result in different chemical potential μ (or concentrations) for vacancies on surfaces under different loads (compression or tension), causing a flux of vacancy (or a flux of matter in the opposite direction) driven by Fick’s law (Fig. 2a). Herring (1950) rigorously formalized the creep problem by considering a small cubic crystal of size *d* that was subjected to pure shear leading to a theoretical constitutive equation:

8 $$\dot{\varepsilon }={A}_{\mathrm{N}\mathrm{H}}\frac{{D}_{\mathrm{s}\mathrm{d}}\Omega }{kT}\frac{\sigma }{{d}^{2}}$$

$\dot{\varepsilon }$ is the steady state creep (strain) rate, Ω is the atomic volume, *d* which characterizes the characteristic length scale of diffusion is the grain size and σ is the shear stress (using the engineering stress would require a multiplication coefficient of 1/3). ${A}_{\mathrm{N}\mathrm{H}}$ is a coefficient whose exact value depends on the geometry of the grains, and ${D}_{\mathrm{s}\mathrm{d}}$ is the self-diffusion coefficient of the rate-limiting species in the bulk. In an idealized scenario wherein a polycrystal is constituted by uniform spherical grains, Herring proposed ${A}_{\mathrm{N}\mathrm{H}}=16$ in the absence of grain boundary sliding and ${A}_{\mathrm{N}\mathrm{H}}=40$ with grain boundary sliding. The creep rate is proportional to stress (linear viscous creep) if the characteristic diffusion length, here the grain size *d* (Fig. 2a), is not a function of stress.

Another diffusion path along grain boundaries was discussed by Coble (1963) as an alternative to lattice diffusion in the bulk. Assuming spherical grains this leads to:

9 $$\dot{\varepsilon }={A}_{C}\frac{{D}_{\mathrm{g}\mathrm{b}}\delta\Omega }{kT}\frac{\sigma }{{d}^{3}}$$

Apart from the introduction of the phenomenological parameter δ (effective grain boundary thickness), the main difference lies in the dependence of the creep rate on 1/d^3^ rather than 1/d^2^, and the dependence of the creep rate on the grain boundary rather than the bulk diffusion coefficient.

In both cases, the creep rate is directly proportional to the diffusion coefficient; therefore, the diffusion coefficient is a fundamental parameter. In compounds, plastic deformation requires that stoichiometric quantities of all elements are transported. It is generally accepted that the process is controlled by diffusion of the slowest species, and it is this species’ diffusion coefficient that is used in expression (4). However, the diffusion phenomenon in ionic compounds can be more complex because the flow of one ion species is influenced by the concentration gradient of other species. This results in coupled diffusion equations that can only be described by the thermodynamics of irreversible phenomena (Regenauer-Lieb et al. 2009). In the case of forsterite, Jaoul (1990) has shown that the net result is a control of diffusional flow by a combination of the slowest diffusion coefficient (Si) times the concentration of the most abundant point defects (Mg vacancies).

An important implicit assumption underlying the model developed by Nabarro and Herring is that grain boundaries act as perfect sources and sinks of vacancies, meaning that the rate of the process is effectively governed by diffusion from one boundary to another. This implies two conditions (Dirichlet boundary conditions) (i) fast diffusion along the grain boundary so the chemical potential is uniform along the concerned segment, (ii) fast interfacial reaction so the grain boundary is instantly equilibrated with the stress. When exchange of vacancies at the grain boundary is finite-rate, the perfect-sink (Dirichlet) condition is replaced by a mixed/Robin condition (Grebenkov 2023). In practice, this induces a threshold stress, meaning that the behavior is no longer linear-viscous, but that of a Bingham solid (Ashby 1969; Artz et al. 1983).

The Nabarro-Herring model provides, in an idealized scheme, the rheological law in the steady-state regime. Since then, efforts have been made to develop micromechanical models of diffusion creep in polycrystals, explicitly modelling the individual grains and their interfaces (Berdichevsky et al. 1997; Villani et al. 2015). The model correctly predicts the strain rate scaling with respect to stress and grain size, *i.e.* $\dot{\varepsilon }=f\left(\sigma ,1/{d}^{2}\right)$, and the overall strain rate is of the same order of magnitude as the one given by the analytical expression (Villani et al. 2015). Strong stress and strain concentrations occur at triple junctions due to increasing strain incompatibilities. Villani et al. (2015) enriched their model with a classical crystal plasticity constitutive framework in order to account for the effects of dislocation creep. This enables the model to describe a smooth transition from diffusion-dominated to dislocation-dominated regimes depending on the level of applied stress (Villani et al. 2015).

A related but distinct case is subgrain creep, in which the relevant diffusion length scale is not the grain size but the spacing between dislocations forming low-angle grain boundaries. In this regime, dislocations act as discrete sources and sinks of vacancies, and their climb mobility directly controls the deformation rate. The discrete nature of these sources introduces threshold stresses, and models predict power-law behavior with stress exponents typically between 2 and 3 depending on the controlling processes (Weertman 1970; Burton 1972; Arzt et al*.* 1983).

###### Nabarro (pure climb) creep

**Fig. 3:**
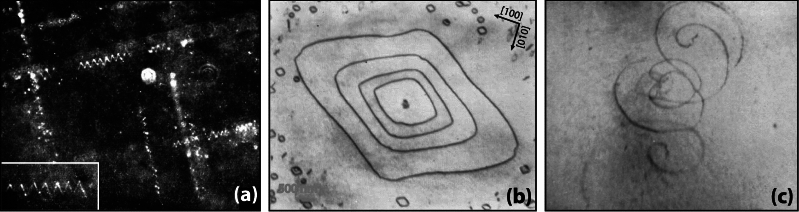
Dislocations as sources and sinks of point defects. **a** Helical dislocations in NaCl resulting from precipitation of vacancies on screw dislocations during cooling from the melt. Magnification 600 × (Amelinckx et al. 1957) **b** Concentric loops formed by climb in Al-3.5%Mg quenched from 550 °C (Westmacott et al. 1962). **c** [0001] dislocation climb spiral sources in magnesium quenched from 400 °C, then annealed 10 min at 120 °C, then quenched in liquid helium, and finally annealed 30 min at 270 °C (Edelin and Levy 1973)

Finally, in pure climb creep (Nabarro 1967; Weertman 1968), deformation is produced by the motion of dislocations moving exclusively by climb within a network that acts as a distributed system of vacancy sources and sinks. The characteristic diffusion distance (*L*_*c*_) is set by the density of dislocations, ρ (Fig. 2c) rather than the grain size. It was discovered very early on that dislocations can act as sinks for vacancies in alloys that have been quenched from high temperatures. These alloys are supersaturated in vacancies after quenching. Heating to a moderate temperature, just enough to enhance diffusion, shows the unique microstructure of climb (Fig. 3 b,c).

The phenomenon is even observed on dislocations close to the screw character, which take on helical shapes (Fig. 3a). These samples (in particular Fig. 3 b,c) showed that it is possible to activate climb dislocation sources, as Bardeen and Herring predicted in 1952. These sources operate on a similar principle to the Frank and Read sources, but with edge dislocations moving by pure climb. In Nabarro creep, strain is produced by edge dislocations exchanging vacancies and moving by pure climb. Steady state emerges from the balance between hardening, which results from the multiplication of dislocations (from the operation of Bardeen-Herring sources), and the recovery, which decreases internal stress due to the dislocation climb. The steady-state strain rate resulting from this mechanism is given by Nabarro (1967):

10 $$\dot{\varepsilon }=\frac{{D}_{\mathrm{s}\mathrm{d}}\mathrm{b}}{\pi kT}\frac{{\sigma }^{3}}{{\mu }^{2}}/\mathrm{ln}\left(\frac{4\mu }{\pi \sigma }\right)$$

**Fig. 4:**
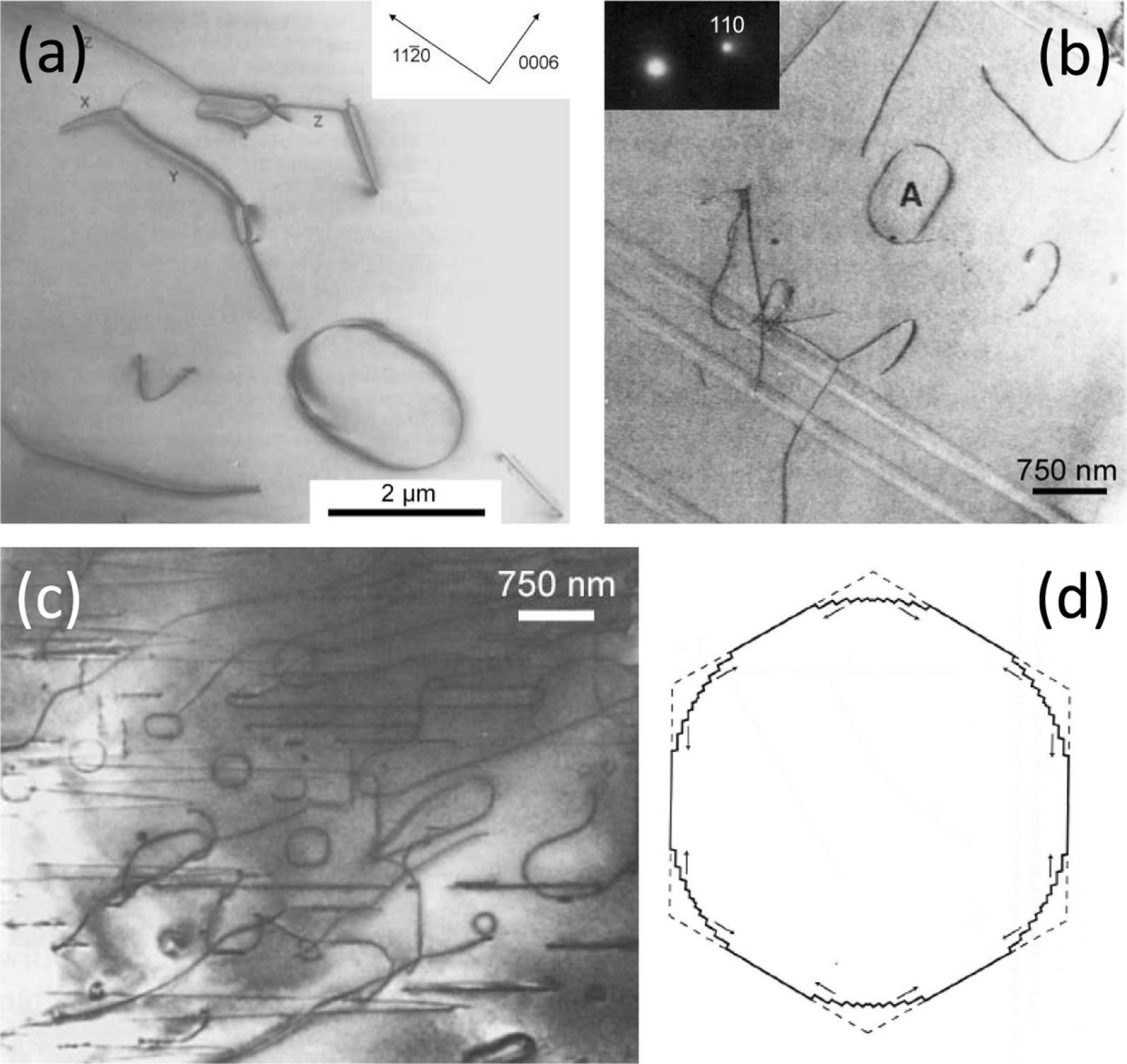
Dislocations in pure climb configurations. **a** Sapphire deformed along [0001]. Dislocations in $\left(1\overline{1 }00\right)$ prism plane foil. TEM bright field (800 kV). Modified after Firestone and Heuer (1976). **b** and **c** KNbO_3_ perovskite (Beauchesne and Poirier 1990), plane of thin foil (001). Prismatic loop A in **b** with a [001] Burgers vector is seen with a residual contrast. **d** In a loop in climb configuration such as this one (where the Burgers vector would be perpendicular to the plane of the figure), the curvatures are geometrically accommodated by jogs

This pure climb creep mechanism has mainly been observed experimentally when dislocation glide was inhibited. A classic example is the case of crystals with hexagonal symmetry compressed parallel to the [0001] axis. Activation of the pure climb mechanism has thus been demonstrated on magnesium single crystals (at 340–630 °C, *i.e.*, 0.66–0.98 T/T_m_) by Edelin and Poirier (1973a,b), beryllium (at 350–1,000 °C, *i.e.*, 0.4–0.8 T/Tm) by Le Hazif et al. (1973), and sapphire (at 1,600–1,800 °C, *i.e.* 0.8–0.9 T/Tm) by Firestone and Heuer (1976). The limited microstructural observations available (Fig. 4 a-c) indicate the presence of loops (frequently termed prismatic) that extend in pure climb planes (See also Firestone and Heuer 1976, Gaboriaud 1981 in Y_2_O_3_ and McLaren et al. 1983 in quartz). With the exception of quasi-crystals (Mompiou et al. 2004), which appear to behave differently, these loops do not appear to exhibit any particular shapes. The climbing loops are curved, which means that they are geometrically saturated in jogs (Fig. 4d). We could therefore refer to them as geometrically necessary jogs. This microstructural feature alleviates the concerns raised by Karato et al. (2025) regarding jog saturation. It is therefore clear that, depending on how it is defined, Nabarro (pure climb) creep can be regarded as either diffusional or dislocation creep, indicating that the commonly used dichotomy between diffusion creep and dislocation creep is, to some extent, artificial and may obscure the continuity of the underlying deformation processes.

These different regimes illustrate that creep behavior emerges from the interplay between diffusion, dislocation motion, and microstructural evolution. Rather than representing fundamentally distinct mechanisms, they correspond to different limiting cases governed by the relative efficiency of atomic transport, dislocation mobility, and recovery processes under given thermodynamic conditions.

### Experiments

The objective here is not to provide a comprehensive review of the experimental approach (see excellent reviews published by Karato et al*.* 2010 or Miyagi 2021 for example), but rather to present the main stages and, above all, to contextualize them within the community’s long-term efforts. Approaching geology from the perspective of experimental science has not always been a natural choice. Following an initial reliance on reasoning and observation, an experimental approach to geology emerged in the nineteenth century, pioneered by Sir James Hall in Scotland and Auguste Daubrée in France. Inspired by James Hutton, Hall carried out the first geological experiments under pressure (already) in 1805, synthesizing rocks such as marble and coal (Hall 1812), meanwhile, Daubrée synthesized diopside, anthracite… (Daubrée 1867). Hall was also responsible for the first experiments in mechanics in geology. By stacking sheets of fabric, then layers of clay subjected to vertical pressure and compressed laterally, Hall succeeded in reproducing and proposing a model for the phenomenon of folding (Hall 1815). Percy Bridgman’s work truly laid the foundations for experimental high-pressure physics, for which he was awarded the Nobel Prize in 1946. This represents a major milestone for our discipline. D.T. Griggs, his collaborator at Harvard, naturally transferred this approach to geophysics. The development of the Griggs rig (Griggs et al. 1960) was a significant breakthrough in the study of rock rheology. To achieve greater measurement accuracy, Paterson (1970) replaced solid confinement with gas confinement in the experimental setup, sacrificing pressure. These two types of machines have undergone technological improvements since then and are still widely used today. However, the field of controlled pressure deformation machines remained limited to around 1 GPa for a very long time. Attempts to exceed this limit consisted of exploring the possibility of using conventional high-pressure devices, such as multi-anvil presses (Durham and Rubie 1997; Karato and Rubie 1997; Weidner et al. 2001; Cordier and Rubie 2001) or diamond anvil cells (DAC, first at room temperature, Meade and Jeanloz 1990; Duffy et al. 1995), to deform samples.

A new milestone was reached with the development of modified multi-anvil presses for controlling plastic deformation with the rotational Drickamer apparatus (RDA, Yamazaki and Karato 2001), the deformation-DIA (Wang et al. 2003), and the DT-CUP (Hunt et al. 2014). Several parameters were crucial to this process. Of course, controlling triaxial stresses at defined strain rates was fundamental. However, it was also necessary to be able to measure stresses and strains. This breakthrough was achieved by combining high-pressure deformation experiments with synchrotron radiation (Chen et al. 2004), which sometimes required the development of transparent anvils. Stress is measured by the displacement of X-ray diffraction peaks (Singh 1993; Karato 2009). In D-DIA, the direct image of the sample allows deformation to be measured.

Temperature control in DAC was more difficult, but this has now been mastered using lasers (Boehler 2000), only on tiny spots, however—*i.e.* with large temperature gradients across the sample, or external heating (Immoor et al. 2020). The development of radial diffraction has made it possible to measure shear strength using lattice strains (Merkel et al. 2002a, b, 2003) and texture development (Wenk et al. 2000, 2014). Using these developments, Immoor et al. (2018) deformed ferropericlase at 1369 K and P up to 65 GPa showing evidence of slip in {100}. Ferropericlase, in a two-phase mixture with bridgmanite was also deformed under lower mantle conditions by Girard et al. (2016) and Miyagi and Wenk (2016). Recently, two studies have focused on davemaoite, one at 14 GPa, 1473 K (Weidner and Li 2024), the other (Immoor et al. 2022) at 1150 K and P up to about 55 GPa, leading to quite different results, davemaoite appearing weaker than MgO or close to bridgmanite. The first deformation of bridgmanite under the P, T conditions approaching those of the lower mantle (Chen et al. 2002) used stress relaxation experiments to infer stress levels from x-ray line broadening. Cordier et al. (2004) deformed bridgmanite at 25 GPa, 1673 K and showed the activation of slip bands containing dislocations which were characterized using the fine structure of x-ray diffraction peaks. Miyagi and Wenk (2016) deformed ferropericlase and bridgmanite aggregates in the DAC using laser heating (1500–1600 K) to follow stress relaxation in the 40–55 GPa pressure range. The first controlled deformation experiments under lower mantle conditions were conducted by Girard et al. (2016), pushing the limits of RDA. Steady-state stress data were acquired up to 20% strain. Despite very high temperatures (> 2000 K), flow stresses of the order of (or greater than) 4 GPa are needed to deform bridgmanite. In the same year, Tsujino et al. (2016) deformed bridgmanite at 25 GPa and 1873 K, analyzing crystal preferred orientations (CPO) and suggesting [001](100) as the dominant slip system. Using a Kawai-type cell, Tsujino et al. (2022) then conducted a series of experiments at pressures of 23–27 GPa and temperatures of 1400–1673 K. The mechanical data at 10⁻5 s⁻1 are fitted with a power law with *n* = 3. Apart from Girard et al. (2016) (see Nzogang et al. (2018)) none of these studies have involved microstructural characterizations.

The study of post-perovskite plasticity relies primarily on advances in laser-heated DAC experiments and radial X-ray diffraction, owing to the very high pressures involved and the non-quenchable nature of the phase. Early experiments on MgGeO_3_ and (Mg,Fe)SiO_3_ suggested dominant slip on (100)-{110} planes and provided the first texture-based interpretations of Dʺ anisotropy (Merkel et al. 2005, 2007). More recent deformation work under near-Dʺ P–T conditions indicates that (001) textures may also form and can reproduce several-percent shear-wave anisotropy (Wu et al. 2017), although agreement with global seismology remains model-dependent. Transformation experiments further show that pPv can inherit strong textures from the precursor perovskite, complicating the distinction between deformation-generated and transformation-induced CPO (Miyagi et al. 2011).

### Scaling laws

The gap between time and space scales and the resulting difficulty in applying experimental data to Earth was immediately recognized. In 1939, Griggs noted: “Other factors acting simultaneously—high temperature, solutions, *and long periods of stress application*—undoubtedly change the strength of rocks…”. Extrapolating experimental data for application to the Earth’s mantle requires rigorous precautions and procedures. The most commonly used tools are parameter normalization and the use of scaling laws.

Let’s start by discussing spatial scales. Certain properties, such as elasticity, can be determined at the unit cell scale. This has enabled the widespread application of ab initio calculations in this field. The first difficulty in scaling up arises when moving from single crystals to polycrystals, for which there is no exact solution, even for elasticity. The situation is even more complex in the ductile regime. Between the atomic scale and the grain scale is a very important intermediate scale: the mesoscopic scale of defects and their microstructure. This intermediate scale considerably impacts rheology. It is also susceptible to changes during deformation. This poses a significant challenge to calculations at the atomic scale, which despite the growing size of accessible systems, remain unable to grasp the complexity of a microstructure. This difficulty can also affect experiments, particularly those carried out at very high pressure in a diamond anvil cell (DAC), where the size of the sample can influence microstructure development. The need to reduce grain size when using small samples at high pressure can also introduce bias by artificially favoring certain mechanisms such as grain boundary sliding.

Rheology is described by constitutive laws that combine variables such as stress, strain and temperature, as well as their derivatives and microstructural parameters (${M}_{i}$) such as grain size and dislocation density, into a function:

11 $$f\left(\sigma ,\varepsilon ,\dot{\sigma },\dot{\varepsilon },P,T,{M}_{i},\dots \right)=0$$

Normalizing some of these parameters to make them dimensionless can reveal master curves and highlight similarities between minerals forming a structural analogous group (Ashby and Brown 1982). Poirier (1985) points out that the best scaling parameter for temperature would be $\mu\Omega /k$ and that the best one for stress would be the cohesive energy per unit cell volume $\Delta {H}_{c}/\Omega$. It is however more convenient to normalize stress by an elastic modulus (Young’s modulus, shear modulus) or by an elastic constant (*e.g.* C_44_), as suggested by dislocation theory, and temperature by the absolute melting temperature T_m_. For strain-rate, Ashby and Brown (1982), clearly having in mind a diffusion-controlled creep process, suggest normalizing by ${D}_{{T}_{m}} /{\Omega }^{2/3}$ where ${D}_{{T}_{m}}$ is the creep limiting diffusion coefficient at the melting temperature.

The concept of structural analogues has proven to be very effective in predicting high-pressure phases (with some exceptions, however: fayalite does not exhibit a wadsleyite-type phase). It has therefore been naturally applied to plastic deformation defining isomechanical groups as suggested by Frost and Ashby (1982). However, the richness and complexity of microstructures and defects mean that the concept of isomechanical groups should be used with caution (Poirier 1988). This has been clearly demonstrated with the perovskite group. The core structure of dislocations is affected by distortions that differ from one phase to another, which prevents the group from being used as an isomechanical group. Nevertheless, this approach is still widely used, particularly when direct experimentation is too difficult, for instance for post-perovskites in Dʺ (*e.g.* Gay et al. 2021) or iron in the inner core (*e.g.* Hunt et al. 2024). The fact that different post-perovskite analogues do not systematically lead to the same CPOs also makes us view this approach with caution.

Equipped with these normalized parameters, Ashby (1972) proposed creating deformation maps that would allow the application fields of different deformation regimes to be compiled and presented synthetically. The fundamental idea is to highlight the concept of *a single dominant mechanism* (more often a creep regime) by comparing the responses (often the strain rate) provided by the different rheological equations for a given set of parameters (Frost and Ashby 1982). The boundaries between domains naturally emerge where two strain rates are equal at a given point. However, Frost and Ashby (1982) were the first to emphasize that "The maps are no better (and no worse) than the equations and data used to construct them". Another important point to bear in mind is ensuring adequate data coverage in the relevant area (see for instance Fig. 14 in Ashby and Verrall 1977).

The use of scaling laws is very powerful but depends on nontrivial assumptions that, in rheology, are sometimes difficult to verify or satisfy. Let us mention a few particularly important ones.

- *Homogeneity—self-similarity*. This assumes that the structure and microstructure of the material, its deformation mechanism, and so on, are self-similar at all scales, or at least within the studied regime. In other words, the mechanism does not change when the size, strain rate, stress, etc. are modified (within the scale regime). This is the condition for power laws with their coefficients to be used. If microstructure or mechanism change, scaling will break or exponents will change.
- *Single dominant mechanism*. Here, the assumption is made that a unique mechanism (not creep regime) dominates, meaning that the rheological response is controlled by a small number of macroscopic variables. This simplifies modelling and makes scaling laws easier to handle and interpret. If multiple mechanisms contribute (possibly at different time or length scales, *e.g.* vacancy diffusion and dislocation glide), then scaling exponents are ‘effective’ only and are likely to vary with conditions (Mitchell et al. 2002). This is already explicitly the case in power-law creep as modelled by Weertman (1955). Moreover, microstructural characterizations of heterogeneous materials (*e.g.* Dimanov et al. 2011) often demonstrate that several mechanisms can be locally concomitant and cooperative in a nonlinear manner due to local and transient stress fluctuations.
- *Stationarity—steady-state*. Assumes that the system has reached a reproducible stage. The risk is evolution of the microstructure or history dependence
- *Negligible boundary/finite size effects*. The sample geometry must be large compared to characteristic lengths. This is particularly important for small samples or when length scales grow, for instance at critical transitions (Kubin and Mortensen 2003; El-Awady 2015).

### The emergence of numerical modeling in the rheology of minerals

The development of multiscale numerical modelling of crystalline plasticity has grown from the foundations of dislocation theory, forming a hierarchy of approaches that link the atomic scale to macroscopic behavior. This development has been possible since the end of the twentieth century due to increased computing power. The range of length scales involved in multiscale modelling spans approximately eight orders of magnitude, from interatomic spacings to hand specimens of rock. Conversely, the characteristic timescales of atomic vibrations and mantle convection differ by about thirty orders of magnitude. Consequently, multiscale modelling involves passing information through three different domains, each with its own specific theoretical approaches and simulation tools: solid state physics, physical metallurgy, and solid-state mechanics. Table 2 summarizes this multiscale framework by linking the main computational approaches to the deformation processes they address, together with the corresponding spatial and temporal scales and the physical parameters transferred across scales.

**Table 2:** Overview of the multiscale framework linking atomic-scale physics to macroscopic rheology

Scale	Methods	Main targets	Elementary processes	Output passed upward
Electronic/atomic	DFT NEB, phonons, defect thermodynamics	defect energies, migration barriers, GSF/γ-surfaces, elastic constants	vacancy formation/migration, Peierls barriers, core energetics	diffusion coefficients, activation enthalpies, Peierls stress, core structures
Atomistic extended	classical potentials, MLIP/MLP MD, NEB, defects thermodynamics	larger defects, finite-T disorder, grain boundaries, complex chemistry	dislocation cores and mobility, GB structure, diffusion pathways	mobility laws, GB energies, pipe/GB diffusion trends, structural transformations
Rare-event atomistic	KMC, accelerated MD, transition-state sampling, activation–relaxation technique	slow thermally activated events inaccessible to MD	diffusion, defect clustering, climb steps, jog evolution	effective diffusivities, event rates, climb mobility
Mesoscale	2D/2.5D/3D DD	collective dislocation behavior	glide, climb, forest hardening, recovery	stress–strain response, steady-state creep, dislocation density evolution
Crystal/aggregate	crystal plasticity, Taylor/Sachs/self-consistent, CPFEM, FFT	single-crystal to polycrystal transfer	texture, slip-system activity, CPO	flow law, anisotropy, texture evolution
Geodynamic	continuum rheology, convection models	planetary-scale consequences	composite rheology	viscosity profiles, strain-rate fields, slab dynamics

For each scale, the table summarizes the main computational approaches, the targeted deformation processes, and the physical parameters or constitutive information passed to the next scale

At the smallest scale, one finds first-principles electronic structure calculations, which most often involve density functional theory (DFT). DFT is a powerful method of solving the Schrödinger equation for a system of N interacting electrons in a tractable way, and is in principle exact (Kohn and Sham 1965). The only approximation arises from our inability to determine the exact exchange–correlation functional. The most common approximations are the local density approximation (LDA) and the generalized gradient approximation (GGA). This step is necessary in order to capture the changes in electronic structure that are induced by pressure. The core structure of dislocations can be obtained based on the Peierls-Nabarro model (Peierls 1940; Nabarro 1947) informed by generalized stacking fault energy calculations (or γ-surfaces, Vitek 1968) which are calculated ab initio. The original limitations of a planar core have been overcome by considering multiple core spreading (Denoual 2004; Metsue et al. 2010). More complex core structures may require direct calculations at the atomic scale based on quantum approaches or on interatomic potentials adjusted on electronic structure calculations (Goryaeva et al. 2015b; Mahendran et al. 2021). This approach currently benefits from the development of Machine-Learning interatomic potentials (Deringer et al. 2019; Yu et al. 2024). Molecular dynamics (MD) simulations of atomic-scale dislocation processes are well-suited to the study of fast athermal events. MD simulations operate at a timescale that corresponds to highly dynamic conditions (strain rates of the order of 10^8^ s⁻1), which are not suited to investigating quasi-static dislocation mechanisms. Atomic diffusion in crystalline solids generally occurs on timescales that are too long to be accessed by conventional molecular dynamics simulations. However, MD simulations have successfully captured faster diffusion pathways, such as grain-boundary diffusion (Riet et al. 2021). Thermally activated dislocation processes, and diffusion processes that are too slow to model using MD, are better modelled using static methods. Nudged Elastic Band methods, for instance, are useful for identifying energy minimum paths, saddle points and activation barriers (*e.g.* Gröger and Vitek 2012). The kinetic Monte Carlo (KMC) method is used to determine the properties of dislocation lines that are subject to competitive processes with different activation probabilities, and to investigate complex diffusion processes that involve multiple jumps with different barriers. It is usually employed in the study of interactions between dislocation points and defects (*e.g.* Landeiro dos Reis et al. 2022). The mesoscale domain is that of the microstructure of defects. At this scale, the aim is to understand the mechanisms that determine the main characteristics of mechanical behaviour:

- the yield stress, which occurs when a significant dislocation density starts to overcome obstacles to its motion
- the flow stress, *i.e.* the stress required to obtain sustained plastic flow at a given plastic strain
- the strain hardening rate (the derivative of the flow stress with respect to the plastic strain)
- and the steady-state strain rate resulting from the application of a given stress.

This can be achieved using Dislocation Dynamics (DD) simulations, which calculate the properties and evolution of dislocation populations based on elastic theory of dislocations. The velocity laws of dislocations are derived from core properties obtained at smaller scales. In the mid-1980s, Lépinoux and Kubin (1987) developed 2D simulations of parallel infinite dislocations with the same Burgers vectors. These simulations were then developed into 3D lattice-based simulations (Kubin et al. 1992). An alternative approach developed by Zbib et al. (1998), among others, is that of nodal simulation.

The largest scale discussed here is governed by solid-state mechanics. At this level, the transition from single crystal to polycrystal—possibly containing multiple phases—occurs. The boundary value problem, which involves the mechanical equilibrium of a body under internal or external fields, can also be addressed at this stage. In polycrystalline plasticity, simple homogenization methods include Taylor’s (1938) uniform strain-rate approximation and Reuss’s (1929) uniform stress approximation. These provide upper and lower bounds, respectively, for the effective yield stress of viscoplastic polycrystals, though the bounds can vary considerably. The self-consistent method (Molinari et al. 1987) models each grain as an inclusion in a homogeneous equivalent medium, leading to a nonlinear stress–strain rate interaction that can be solved with Newton’s method. Recently, increased computational power has enabled full-field simulations of polycrystals via the finite-element method (FEM), while an alternative approach using the fast Fourier transform (FFT) was introduced by Moulinec and Suquet (1994).

## Tracing the evolution of multiscale modeling in mantle minerals rheology

### The epic of (ferro)periclase

There are two reasons why we paid particular attention to periclase (MgO). Firstly, it is present (combined with iron) in the lower mantle. It is also a stable oxide at ambient pressure and has been the subject of considerable study, providing a very robust basis for comparing our models. In terms of crystal chemistry complexity, MgO also represents an intermediate state between metals, for which numerical models have generally been developed, and silicates. Just as the Drosophila fly is used as a model organism in genetics, we used periclase as a model mineral to develop our methods. MgO is one of the first minerals for which atomic-scale modelling of defects such as dislocations (Puls and Norget 1976; Woo and Puls 1977; Rabier and Puls 1989) and grain boundaries (Harris et al. 1996; Hirel et al. 2018) has been undertaken. MgO crystallizes in the rock-salt (NaCl) structure also called B1. A large body of experiments (see Amodeo et al. 2011) have shown that plastic deformation proceeds by glide of $1/2\langle 110\rangle$ dislocations in the {110} or {100} planes.

#### Dislocation core structures

The current understanding of the core structure of dislocations in MgO is the result of nearly 40 years of research employing a variety of techniques that have evolved in tandem with advances in computing technology. The first models of $1/2\langle 110\rangle$ dislocation cores, first edges (Puls and Norget 1976; Rabier and Puls 1989), then screws (Watson et al. 1996), were made on cluster-based embedded models. Secondly, the core structure was addressed using the Peierls-Nabarro (PN) model. This model is often referred to by the Peierls stress formula (Joós and Duesbery 1997), which for an edge dislocation reads as follows:

12 $${\sigma}_{P}=\frac{2\mu }{\left(1-\nu \right)}\mathrm{exp}\left(-\frac{4\pi \zeta }{b}\right)$$

where ζ is the dislocation half-width, μ the shear modulus, ν the Poisson ration and *b* the Burgers vector modulus. However, this is obtained at the cost of making the very strong assumption of a sinusoidal restoring force. This assumption is rarely verified and does not permit the original model to be used for quantitative predictions. However, using Vitek’s (1968) proposed generalized stacking faults (GSFs) in the PN model enables a much more realistic representation of dislocation cores, provided they satisfy the model’s fundamental assumptions regarding planar core spreading. These models (Miranda and Scandolo 2005; Carrez et al. 2009) enabled the creation of realistic dislocation core models, confirming the spreading suggested by the initial calculations (Puls and Norget 1976; Rabier and Puls 1989). They also confirmed the well-established experimental finding that glide in {110} is much easier than in {100}. As the GSF calculations that inform the PN model can be performed using first-principles, they enable the effects of pressure on the core structure to be taken into account (Carrez et al. 2009). The following developments resulted in a modification of the PN model: the Peierls-Nabarro-Galerkin (PNG) method. This method relaxes the assumption of planar core spreading by considering the possibility of spreading in several planes (Amodeo et al. 2012). Finally, Carrez et al. (2015) performed fully atomistic calculations of dislocation cores based on first-principles calculations using periodic arrangements of dislocation quadrupoles. The cores computed according to the PNG model (Amodeo et al. 2012) were found to perfectly match the atomistic configurations of Carrez et al. (2015). An important result of these calculations was the prediction of a pressure-dependent change of slip systems, with $1/2\langle 110\rangle \left\{100\right\}$ slip gradually becoming easier than $1/2\langle 110\rangle \left\{110\right\}$ slip between 30 and 60 GPa. This theoretical prediction corroborates a hypothesis proposed by Karato in 1998 and was recently validated through experimental research conducted by Immoor et al. (2018).

#### Dislocation glide mobility

**Fig. 5:**
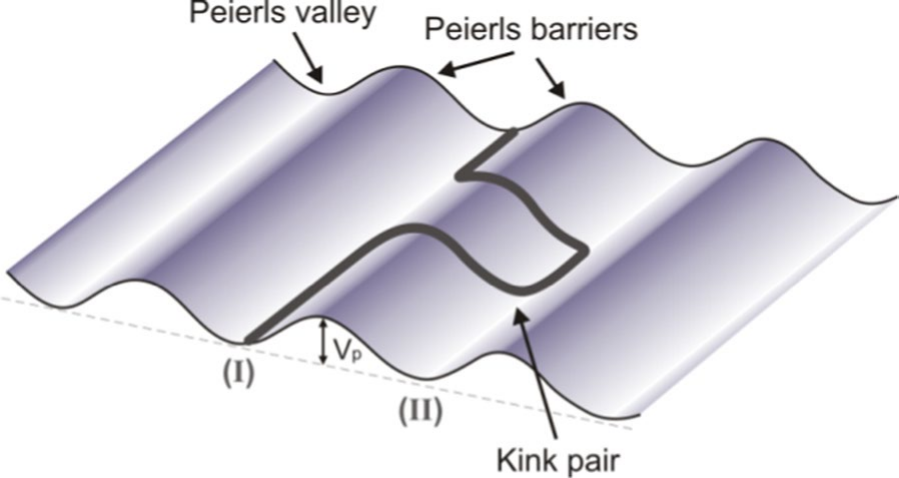
Thermal activation of dislocation glide. Schematic illustration of the kink-pair mechanism which allows the dislocation line to pass from one Peierls valley to the next one at finite temperature

**Fig. 6:**
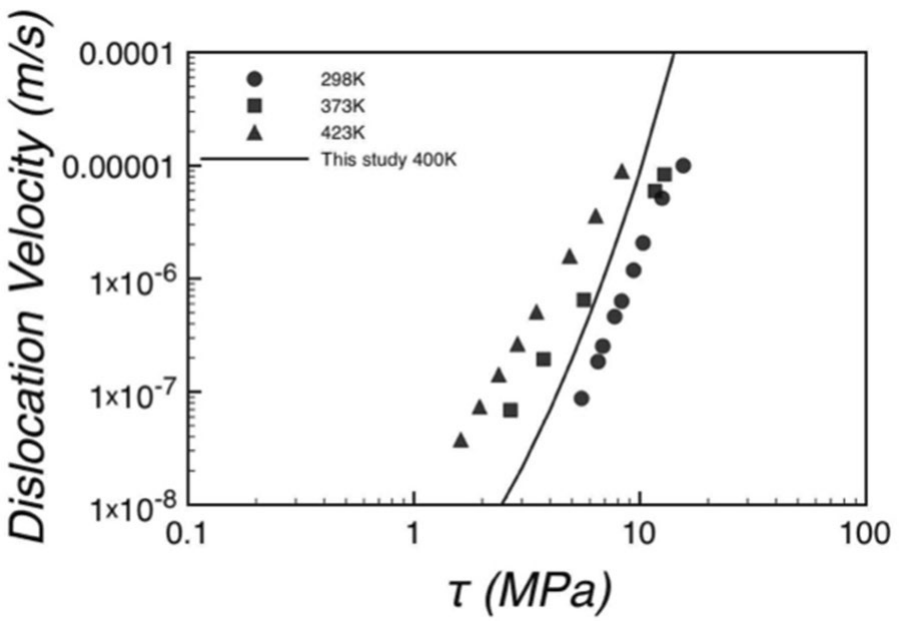
Dislocation velocity of the ½ < 110 > screw dislocation in MgO. Comparison between the velocity predicted by Eq. 13 and the average velocities of screw dislocations measured experimentally by Singh and Coble (1974)

The core structure of dislocations is fundamental to understand their mobility. The critical information is the Peierls stress, which is a measure of the intrinsic resistance to glide opposed by the crystal lattice. At room pressure, the Peierls stresses for $1/2\langle 110\rangle \left\{110\right\}$ dislocations are 150 MPa for screw and 80 MPa for edge components. For $1/2\langle 110\rangle \left\{100\right\}$, the Peierls stresses are 1600 MPa and 300 MPa for screw and edge components respectively. MgO is therefore a mineral with relatively high crystalline friction. Below the athermal temperature *T*_*a*_ (see Fig. 1), Peierls barriers are overcome by the thermally activated kink-pair mechanism (Fig. 5). In the case of undissociated dislocations with cores in glide configuration, there exist several models that can be used to calculate the critical nucleation enthalpy of kink-pairs. One of them, the elastic interaction model, was applied by Amodeo et al. (2011) to MgO. The critical configuration (of enthalpy $\Delta {H}^{*}$) appears to have widely spaced (${w}_{c}$) and spread-out kinks, which suggests a low migration enthalpy barrier. Therefore, the nucleation of kink pairs is the limiting stage of gliding. Based on the Guyot and Dorn (1967) and Kocks et al. (1975) formalisms, it is possible to derive a velocity law for dislocations (here the rate-limiting screws) in MgO (Amodeo et al. 2011):

13 $$v\left(\tau ,T\right)=\frac{a{\prime}{\nu}_{D}bL}{{2w}_{c}^{2}}\mathrm{exp}\left(-\frac{\Delta {H}^{*}\left(\tau \right)}{kT}\right)\text{ with} \Delta {H}^{*}\left(\tau \right)={\Delta {H}_{0}\left(1-{\left(\tau /{\tau}_{P}\right)}^{p}\right)}^{q}$$

for which all parameters are accessible by calculation. $a{\prime}$ is the periodicity of the Peierls potential, ${\nu}_{D}$ is the Debye frequency, *b* is the Burgers vector modulus, *L* is the dislocation length, $\Delta {H}_{0}=\Delta {H}^{*}\left(\tau =0\right)$ corresponds to twice the energy ${U}_{k}$ of an isolated kink, τ is the resolved shear stress, and *p* and *q* are parameters adjusted on the parameters of the critical configuration. The results of this calculation compare favorably with the scarce experimental data on dislocation velocities (Fig. 6). It should be noted that this modeling approach is more complex than a direct MD calculation, but that the latter would not have access to thermally activated phenomena whose characteristic times would be longer than those of MD simulations, and therefore cannot be applied to simulate low velocities.

#### Above ***T***_***a***_: dislocation interactions

**Fig. 7:**
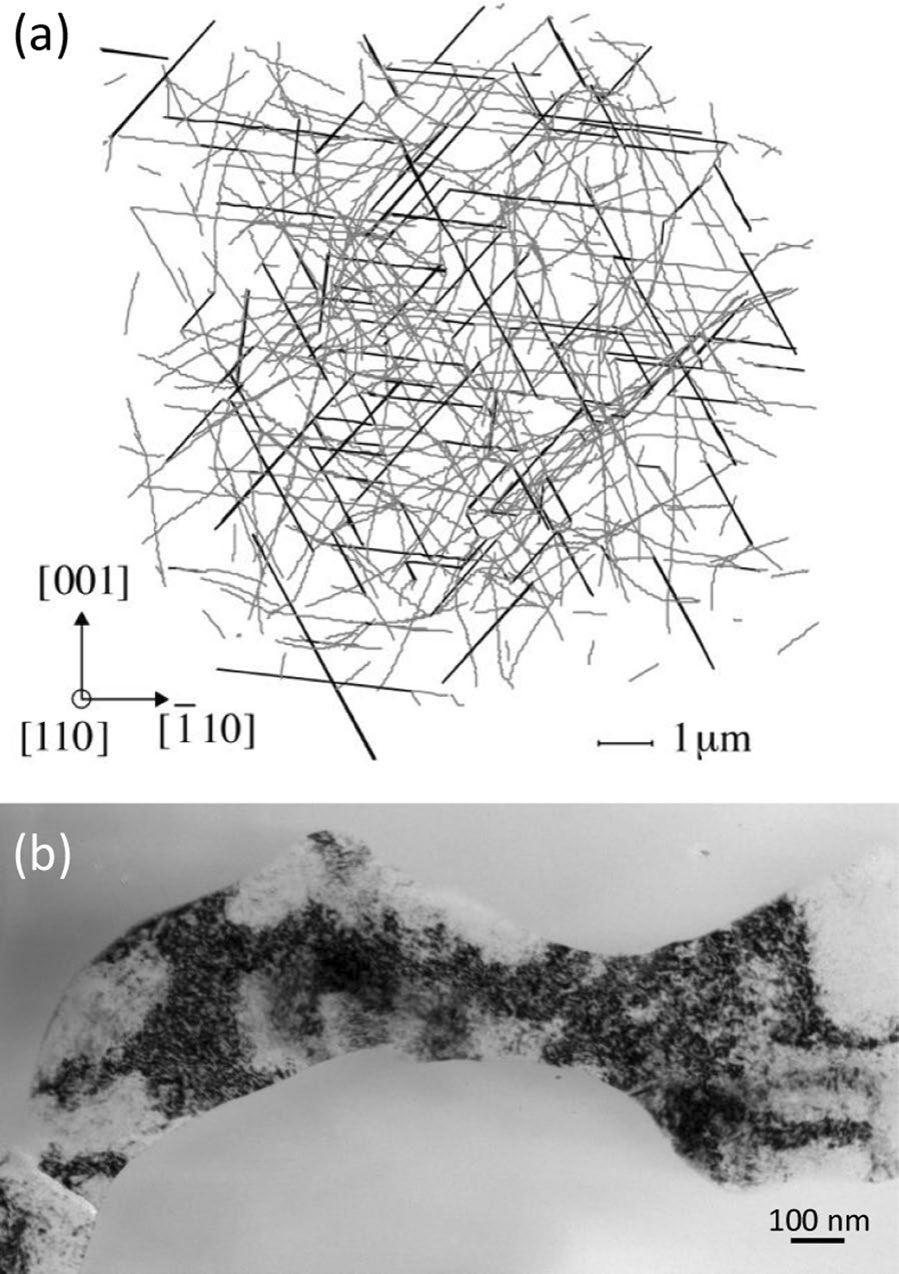
Dislocation (forest) hardening in (ferro)periclase. **a** Example of a dislocation microstructure for simulating dislocation interactions within the $1/2\langle 110\rangle \left\{110\right\}$ slip systems (Amodeo et al. 2014) **b** Ferropericlase grain deformed at 27 GPa and 2130 K (specimen Gamma 21, Girard et al. 2016). The matrix is amorphized bridgmanite). The dislocation density is very high, making it impossible to resolve individual contrasts. It is estimated at 10^15^ m.^−2^

Above the athermal temperature *T*_*a*_, lattice friction vanishes and the intrinsic velocity of dislocations is limited only by a viscous drag. However, the effective velocity actually depends on other interactions, particularly the interaction between dislocations themselves. Before considering (in the case of creep, Sect. 4.1.5) how dislocations can be freed from these interactions through diffusive processes, let us try to describe them. Two complementary approaches are possible. One approach involves studying the elementary interactions resulting from the crossing of two dislocations belonging to different slip systems. This lends itself well to elastic calculations, which can be either analytical or numerical using dislocation dynamics (DD). This enables the possible interactions (*e.g.* junction formation, crossed states and repulsive interactions) and their frequencies to be listed (Amodeo et al. 2014). The other approach consists of performing large-scale DD simulations that allow all these interactions to be taken into account collectively (Fig. 7a).

It is thus possible to model the hardening of the dislocation forest^3^ resulting from these interactions, which can be described by an expression of Taylor’s law in the form:

14 $${\tau}_{f}=\mu b\frac{\mathrm{ln}\left(1/b\sqrt{\beta {\rho}_{f}}\right)}{\mathrm{ln}\left(1/b\sqrt{\beta {\rho}_{ref}}\right)}\sqrt{\beta {\rho}_{f}}$$

where ${\rho}_{f}$ is the forest dislocation density and ${\rho}_{\mathrm{r}\mathrm{e}\mathrm{f}}$ is the reference dislocation density, and β is a forest strengthening coefficient which is calibrated with the DD simulations (Fig. 7a). This modelling enabled us to accurately interpret the stress values (∼ 0.9 GPa) measured in the ferropericlase grains from the experiments conducted by Girard et al. (2016), based on the microstructural characterizations performed on these samples (Nzogang et al. 2018), in particular, from the determination of the dislocation density (${\rho}_{f}\sim {10}^{15}{\text{ m}}^{-2}$ see Fig. 7b). This forest hardening is very effective. The stress would only be much lower (25 MPa) for a dislocation density ${\rho}_{f}\sim {10}^{12}{\text{ m}}^{-2}$.

#### From the glide of a single dislocation to the deformation of a crystal, then of a polycrystal

The simplest approach is to use Orowan’s law (Eq. 5) and combine it with the velocity law (Eq. 13) to arrive at an equation of the form:

15 $$\dot{\varepsilon }=\sqrt{\rho }\frac{a{\prime}{\nu}_{D}{b}^{2}}{{2w}_{c}^{2}}\mathrm{exp}\left(-\frac{\Delta {H}^{*}\left(\tau \right)}{kT}\right)$$

**Fig. 8:**
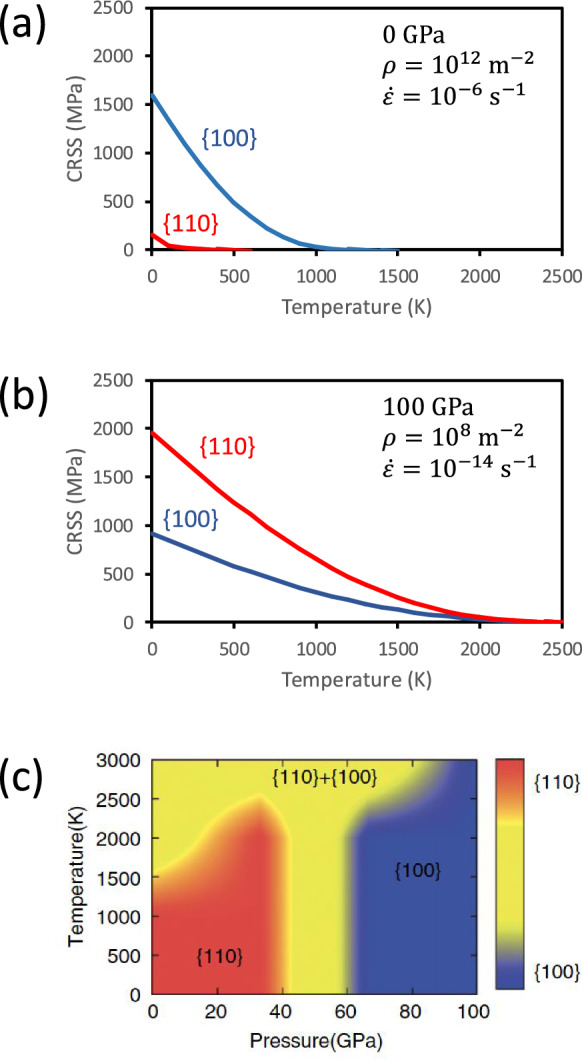
Critical resolved shear stresses (CRSS)of the two main slip systems 1⁄2〈110〉{110} and 1⁄2〈110〉{100} in MgO, as predicted by modelling. **a** Conditions representative of laboratory experiments. **b** pressure and strain-rate representation of lower mantle conditions with a dislocation density adjusted to the strain-rate. Note the inversion of slip systems between **a** and **b**. **c** P, T domains of prevalence of different slip systems calculated for a dislocation density of 10^12^ m^−2^ and a strain rate of 10^–4^ s^−1^. The yellow domain defines (P,T) conditions where CRSS for the both slip systems are of the same magnitude

A key advantage of this formulation is that it explicitly relates the stress τ to temperature at a given strain rate, such that the strain rate becomes an input parameter rather than requiring extrapolation (Cordier et al. 2012). The stress τ in (15) corresponds to dislocation glide in the thermally activated regime (*i.e.* below *T*_*a*_) and can be assimilated to the critical resolved shear stress (CRSS). This approach describes the onset of plasticity, *i.e.*, the conditions under which specific slip systems become activated. It does not, however, account for the subsequent evolution of the microstructure and its feedback on rheology during large strains. Incorporating the results of our calculations as a function of pressure nevertheless allows us to capture, in a physically grounded way, the combined influence of pressure, temperature, and strain rate on the activation of slip systems (Amodeo et al. 2012; Cordier et al. 2012). In particular, the extent of the thermally activated glide domain and the relative ease of $1/2\langle 110\rangle \left\{110\right\}$ and $1/2\langle 110\rangle \left\{100\right\}$ slip systems emerge clearly (Fig. 8). Recent high-pressure deformation experiments on MgO using rotational diamond anvil cells (Ishimori et al. 2025) provide an important point of comparison. These experiments, performed up to ~ 120 GPa and ~ 1000 K, probe deformation at large strains and reveal a dominance of $1/2\langle 110\rangle \left\{100\right\}$ slip under high-pressure and high-temperature conditions. This observation is consistent with our predictions of a transition toward $1/2\langle 110\rangle \left\{100\right\}$ glide at high pressure, and even suggests that this regime may extend over a broader range of conditions, particularly at elevated temperatures. The apparent difference in the extent of the stability fields likely reflects the different nature of the approaches: while our calculations determine the onset of slip-system activation, the experiments of Ishimori et al. (2025) document deformation at large strains, where microstructural evolution and interactions between slip systems may further modify the dominant deformation modes. This comparison highlights the complementarity between mechanism-based modeling and high-pressure deformation experiments. The studies of Amodeo et al. (2012) and Cordier et al. (2012) thus provide a first-principles description of a key component of mineral plasticity, dislocation glide, under lower-mantle conditions, including pressure, temperature, and strain rate, without the need for extrapolation.

Another, more sophisticated approach consists of performing DD calculations (Fig. 7a). These calculations take into account both dislocation velocities and interactions (Amodeo et al. 2011). They enable a more detailed comparison with experimental results, thus allowing the model to be validated (Amodeo et al. 2011). They have also made it possible to model forest hardening (Amodeo et al. 2014; see above). A simpler and more numerically tractable alternative, which will enable the transition to creep as we will see below, is the implementation of 2.5D DD (Reali et al. 2017). This is a 2D formulation of DD with local rules implemented to reproduce the 3D simulations of DD (against which the model is benchmarked).

**Fig. 9:**
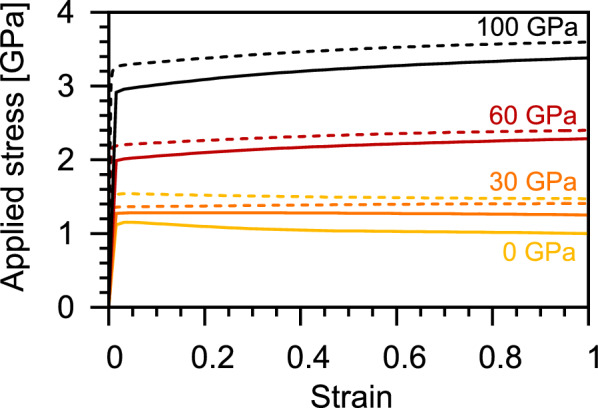
Plastic deformation of MgO polycrystals under confining pressure: modelling results. Stress strain curves of MgO polycrystals compressed under 0, 30, 60 and 100 GPa hydrostatic pressures using the crystal plasticity finite element method (full lines) and the Taylor model (dashed lines). Temperature is 300 K and strain rate is 10^–4^ s^−1^. Amodeo et al. (2016) with permission. The hardening effect of pressure is clear

The transition between elementary mechanisms (dislocation glide) and polycrystalline plasticity was achieved by Amodeo et al. (2016). Two approaches were followed. The simplest is Taylor’s, which assumes a uniform strain throughout the polycrystal. Finite element modeling based on crystalline plasticity was also developed which provides a more realistic prediction by accounting for pair-wise interaction of adjacent grains. Within each grain, the plastic shear emulated by the Schmid tensor results from the Orowan equation informed by the velocity laws presented above (Amodeo et al. 2016). Although these calculations have so far been performed only at room temperature, they already demonstrate that first-principles based, multiscale modeling can predict the flow laws of polycrystals (Fig. 9) and reproduce texture evolutions consistent with experimental observations (Merkel et al. 2002b).

#### Creep

Karato et al. (2025) are right to note that the model developed by Cordier et al*.* in 2012 primarily describes glide-controlled creep and does not incorporate all high-temperature deformation mechanisms. However, subsequent studies have extended this framework, demonstrating that the multiscale modeling of MgO rheology has continued to evolve well beyond 2012.

A new ingredient to consider here is dislocation climb, which is controlled by the diffusional flux of vacancies. The interaction between charged vacancies (of both Mg and 0) and $$1/2\langle 110\rangle \left\{110\right\}$$ edge dislocations has been calculated by molecular statics and the elastic dipole method (Landeiro dos Reis et al. 2021). The edge dislocation core, within a region which extends over several Burgers vectors across the glide plane, exerts a strong attraction to vacancies, especially anionic (oxygen) ones. Therefore, edge dislocations in MgO act as strong sinks for vacancies. The oxygen vacancies also diffuse easily along the dislocation core, making pipe diffusion particularly efficient for oxygen defects (Landeiro dos Reis et al. 2022). Jogs, which enable dislocation climb through vacancy absorption or emission, have been characterized atomistically in MgO by Zhai et al. (2020), who showed that along $${\raise0.7ex\hbox{$1$} \!\mathord{\left/ {\vphantom {1 2}}\right.\kern-0pt} \!\lower0.7ex\hbox{$2$}}\left\langle {110} \right\rangle \left\{ {110} \right\}$$ edge dislocations, elementary jogs of height $${\raise0.7ex\hbox{$1$} \!\mathord{\left/ {\vphantom {1 4}}\right.\kern-0pt} \!\lower0.7ex\hbox{$4$}}\left[ {110} \right]$$ spread in {111} planes and carry a half-charge (± q/2), whereas neutral super-jogs of height $${\raise0.7ex\hbox{$1$} \!\mathord{\left/ {\vphantom {1 2}}\right.\kern-0pt} \!\lower0.7ex\hbox{$2$}}\left[ {110} \right]$$ can also form.

Modelling creep presents several difficulties. One major issue is the requirement to consider two extra mechanisms (and their interactions): vacancy diffusion and dislocation climb. Another significant challenge is that these mechanisms potentially operate on very different timescales. To develop a tractable creep model, Reali et al. (2017) used 2.5D DD simulations. A 2D formulation, in which dislocations are treated as straight, infinite lines, is sometimes sufficient to answer some fundamental questions in plasticity. The limitations of this formulation can be overcome by introducing local rules that mimic important 3D mechanisms as closely as possible (Gómez-García et al. 2006). Once this has been achieved, 2.5D DD simulations are very well adapted to coupling glide and climb (Davoudi et al. 2012; Keralavarma et al. 2012). The 2.5D DD model of MgO (Reali et al. 2017) has been benchmarked against experimental results and 3D-DD simulations (Amodeo et al. 2011) in the low-temperature regime (T ≤ 600 K), where thermally activated glide of dislocations occurs. In an intermediate regime (T = 1000 K), where plasticity is dominated by dislocation–dislocation interactions, it has been benchmarked against 3D-DD simulations (Amodeo et al. 2014). Finally, in the high-temperature creep regime (1500 ≤ T ≤ 1800 K), the 2.5D DD model of MgO (Reali et al. 2017) predicts a steady-state creep regime characterized by a stress exponent close to 3 and a creep activation enthalpy close to the oxygen self-diffusion enthalpy used in the model (Yoo et al. 2002). The model’s results are consistent with the available experimental data on MgO at ambient pressure over a wide temperature range, confirming its ability to describe MgO rheology.

It should be noted that, in this model, the diffusion coefficient of the limiting species—a parameter of prime importance—does not come from our multiscale modelling chain. Rather, it is data taken from the literature. As studied by Reali et al. (2017), the value has a very strong influence on the model results. When applying this model to mantle conditions, selecting the appropriate diffusion data is critical. Diffusion in MgO has been studied extensively both experimentally and by first-principles methods, and reviewed by Van Orman and Crispin (2010). In both synthetic and natural periclase crystals, trivalent cations such as Fe^3+^ and Al^3+^ are typically the predominant impurities, and are charge-balanced by (extrinsic) cation vacancies. Oxygen vacancies, on the other hand, are intrinsic, and oxygen diffusion is much slower than cation diffusion. Intrinsic oxygen diffusion is therefore expected to be the rate-limiting step for creep in periclase. The diffusivity of oxygen does not vary with the concentration of trivalent cations in MgO, indicating that neutral, bound Mg-O vacancy pairs rather than positively charged oxygen vacancies control oxygen diffusion. Because the concentration of these intrinsic Schottky defects is so low, even at high temperatures, oxygen volume diffusion in periclase is very slow and difficult to measure experimentally without enhancement due to dislocations or other structural defects. Nevertheless, careful experiments on CVD-grown MgO with very low defect density yield O diffusion results at atmospheric pressure and high temperatures that are in reasonable agreement with theoretical calculations for diffusion via intrinsic Mg-O vacancy pairs (Yang and Flynn 1994).

**Fig. 10:**
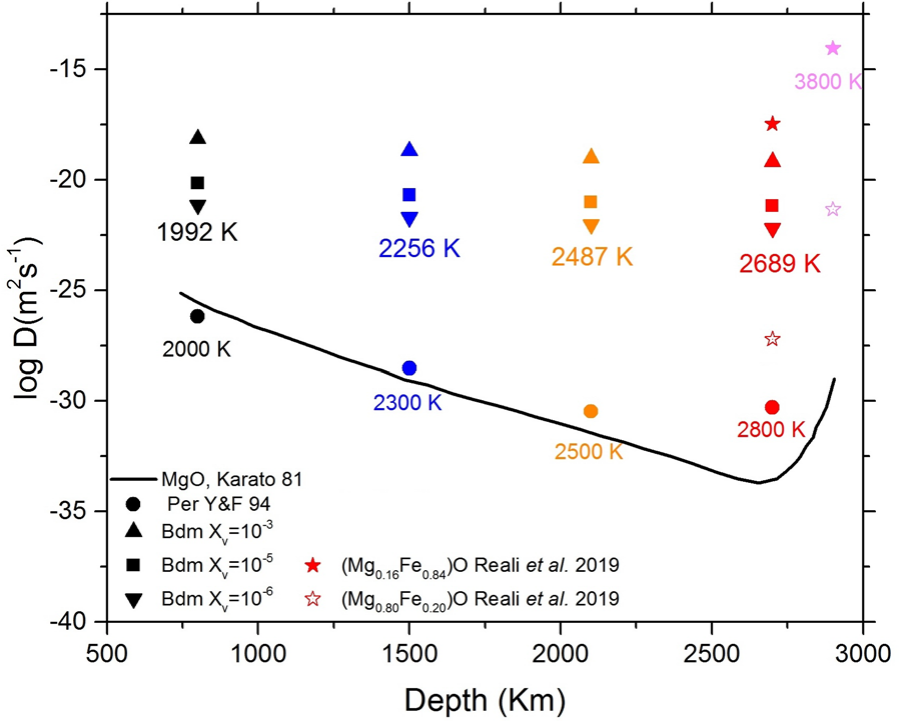
Diffusion coefficients in bridgmanite (Bdm), periclase (Per), and magnesiowüstite along a representative lower-mantle geotherm. For bridgmanite, the values correspond to cation diffusion (Mg and Si considered equivalent) calculated for a range of vacancy concentrations (X_v_ = 10^–3^−10^–6^). For periclase and magnesiowüstite, oxygen self-diffusion is shown, as oxygen diffusion is expected to control creep. Y&F 94 refer to the pressure dependence of Ita and Cohen (1997) adjusted on experimental data from Yang and Flynn (1994). Data for magnesiowustite were obtained by scaling oxygen self-diffusion coefficients with the homologous temperature $T/{T}_{m}$ (Reali et al. 2019b)

To apply the creep model to MgO at mantle pressures and temperatures, Cordier et al. (2023) relied on the calculated pressure dependence for oxygen self-diffusion, as determined by Ita and Cohen (1997), which align with the room-pressure data of Yang and Flynn (1994). In agreement with the calculations of Karato (1981), these calculations demonstrate the significant impact of pressure on oxygen diffusion in MgO (Fig. 10). This results in extremely slow creep rates for MgO under lower mantle conditions, which are slower than those for bridgmanite under the same conditions.

This extremely slow diffusion of oxygen has another important consequence. In multiscale modeling, it is often observed that, as the modeling scale increases, certain microscale parameters become unidentifiable or effectively irrelevant to the macroscopic response. In the present case, the waiting time at obstacles controlled by diffusion and climb is very long such that the dislocation glide velocity, whether thermally activated or athermal, has no influence on the overall creep rate.

The scarcity of diffusion data under lower mantle conditions is a critical limitation to our understanding of rheology. This is the difficulty we encounter in developing the model toward more realistic situations, for example by taking into account the iron content of ferropericlase. Although, as noted above, O diffusivity is insensitive to the presence of Fe^3+^ or other trivalent impurities, Fe substitution at higher concentrations will change the Schottky defect formation and migration energies and hence will influence the rate of O diffusion. Although O diffusion in (Mg,Fe)O solid solutions has not been studied extensively, there are anion diffusion data for wüstite (Fe_1-x_O) and several other oxides and halides with the rock salt (B1) structure, which have very similar values at the same homologous temperature $T/{T}_{m}$ (Reali et al. 2019b). We used this homologous temperature scaling to predict O diffusion and creep rates in to predicting the rheology of magnesiowustite (*i.e.* for high iron content) under deep very lower mantle conditions, we used the scaling of oxygen self-diffusion coefficients with the homologous temperature $T/{T}_{m}$ (Reali et al. 2019b).

### The saga of bridgmanite

Unlike MgO, bridgmanite is not stable at ambient pressure, so we could not validate our models using well-established experimental knowledge. However, bridgmanite belongs to the large class of perovskites, some of which, such as tausonite (SrTiO_3_), have been the subject of numerous high-quality studies. While we did not intend to adopt an analogous materials approach, other perovskites present the same challenge in terms of crystal chemistry complexity and are therefore relevant for testing the validity of our models.

As with MgO, our initial approach was to model the dislocation core using the Peierls–Nabarro model. This model has performed excellently in modelling the dislocation core in both SrTiO_3_ (Ferré et al. 2008) and CaTiO_3_ (Ferré et al. 2009). We were therefore able to extend this approach to bridgmanite using the methodology established for MgO. This involved applying the PN model, which is informed by first-principles calculations of GSFs, to account for the effect of pressure. The first models of dislocation cores in bridgmanite were thus obtained at pressures of up to 100 GPa (Carrez et al. 2007a; Ferré et al. 2007). These models demonstrate the significant impact of orthorhombic distortions and octahedra tilting on the spreading of dislocation cores. This study was continued using the PNG model, which provides a more reliable description of the spreading of screw dislocation cores (Gouriet et al. 2014). It made it possible to identify the easiest slip systems in bridgmanite on which to focus further studies: [100](010) and [010](100). These dislocations were studied in greater depth in 2014 by Hirel et al*.* based on supercell molecular static calculations.

**Fig. 11:**
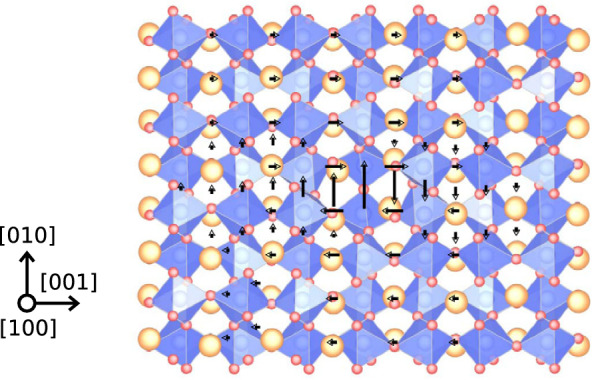
Atomic configuration of the $\left[100\right]$ screw dislocation in MgSiO_3_ bridgmanite viewed in its (010) glide plane. Mg ions appear in yellow, Si in blue, and O in red. (Kraych et al. 2016a)

**Fig. 12:**
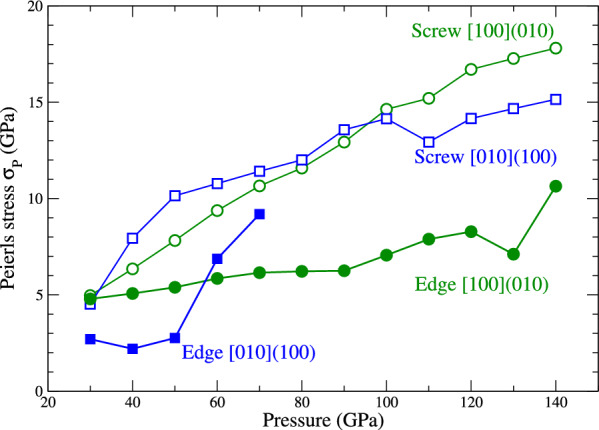
Pressure dependence of lattice friction for edge and screw dislocations in the [100](010) and [010](100) slip systems of MgSiO_3_ bridgmanite (Hirel et al. 2014)

The core structures observed in atomistics models corroborate the findings of the PNG model. Notably, all dislocations are found to have a compact core (Fig. 11). Bridgmanite differs from other perovskites in this respect, which explains why they do not form an isomechanical group. However, the PNG model underestimates Peierls stresses because it does not describe local effects due to ionic charges and electrical polarization. A key finding of this study is the significant lattice friction experienced by dislocations. This is due to orthorhombic distortions, which increase stacking fault energies and result in narrower cores. As these distortions are amplified by pressure, bridgmanite exhibits a dramatic increase in lattice friction within the lower mantle pressure range (Fig. 12). This pressure effect results in a steady monotonic increase in lattice friction on screw dislocations (Hirel et al. 2014). Edge dislocations behave differently. Although they appear less sensitive to pressure, their lattice friction increases sharply at a certain pressure, to the point of inhibiting glide (Fig. 12, this effect is most visible on [010](100) edge dislocations).

To understand this unusual behavior, let us return to SrTiO_3_, which exhibits a surprising ductile–brittle transition as a function of temperature (Gumbsch et al. 2001). In this oxide, the dislocation core spontaneously transitions from a gliding configuration (with the core spreading in the slip plane) to a sessile configuration (with the core spreading in the climb plane) as the temperature increases (Hirel et al. 2016a,b, 2025). This type of change in the structure of the core, which has already been observed in oxides (Gaboriaud 1980, 1981), inhibits glide and explains the brittle behavior of SrTiO_3_ at high temperatures (Gumbsch et al. 2001). The same type of core transformation is also observed in bridgmanite (Hirel et al. 2016b), but due to the narrow cores, the transformation occurs more easily under pressure alone, independently of temperature. This leads to the inhibition of the [100](010) slip system at around 140 GPa, and of the [010](100) slip system at around 60–70 GPa (Fig. 12).

Despite this result already casting doubt on the ability of bridgmanite to deform by dislocation glide in the lower mantle, we continued modelling to include thermal activation. The compact structure of the dislocation cores suggests the use of modelling based on the kink-pair mechanism, as with MgO. Once again, we validated our approach using SrTiO_3_, for which accurate data on lattice friction at low temperatures is available. As demonstrated by Carrez et al. (2010), the kink-pair mechanism model perfectly describes the thermal activation of glide in SrTiO_3_. It has then been applied to MgSiO_3_ bridgmanite by Kraych et al. (2016a). To this end, the Peierls potential was accurately calculated for the [100](010) slip system using atomistic calculations and the nudged elastic band (NEB) method. With Peierls stress confirmed at 4.9 GPa at P = 30 GPa, MgSiO_3_ bridgmanite is a mineral with high lattice friction. Next, the shape and enthalpy (${H}_{k}$) of an isolated kink were calculated. Finally, based on the elastic interaction model, the stress dependence of the critical kink-pair nucleation enthalpy ($\Delta {H}_{k}^{*}$) was calculated (Kraych et al. 2016a). These calculations were extended to 60 GPa for both [100](010) and [010](100) slip systems (Kraych et al. 2016b). Having established these fundamental parameters at the atomic scale, we can deduce, as for MgO, a velocity law for gliding dislocations. This, when combined with Orowan’s law, enables us to calculate the evolution of the CRSS with temperature for a given strain rate.

**Fig. 13:**
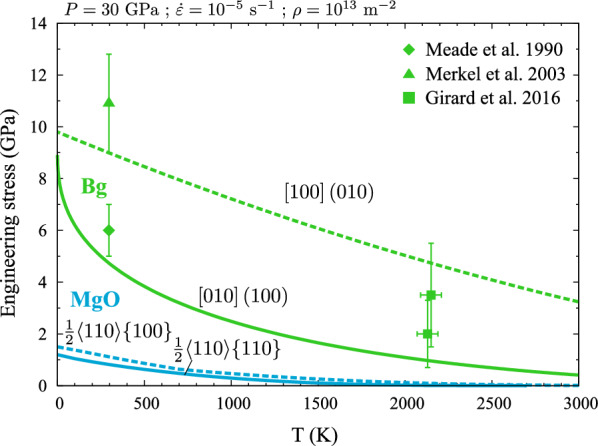
Dislocation glide in MgSiO_3_ bridgmanite under laboratory conditions: P = 30 GPa, $\dot{\varepsilon }={10}^{-5} {s}^{-1}$ (Kraych et al. 2016b). To facilitate comparison between the model results (lines) and experimental data (symbols), the CRSS values have been multiplied by 2 to account for the Schmid factor. Numerical predictions for MgO, calculated under the same conditions as described above, are also shown for comparison. The values for two slip systems closely match the experimental results, suggesting that the high stresses observed experimentally are indeed due to the strong lattice friction opposing dislocation motion in bridgmanite

Figure 13 shows the result of such calculations with parameters chosen for a comparison with experimental results: P = 30 GPa, $\dot{\varepsilon }$=10^–5^ s^−1^ and a dislocation density ρ=10^13^ m^−2^. The orders of magnitude derived from this simple dislocation slip model are in excellent agreement with experimental values. These results confirm that the crystal structure of MgSiO_3_ bridgmanite exerts very strong lattice friction on dislocation glide at 30 GPa, even at very high temperatures. Indeed, the athermal glide regime is never reached for this mineral under these conditions.

**Fig. 14:**
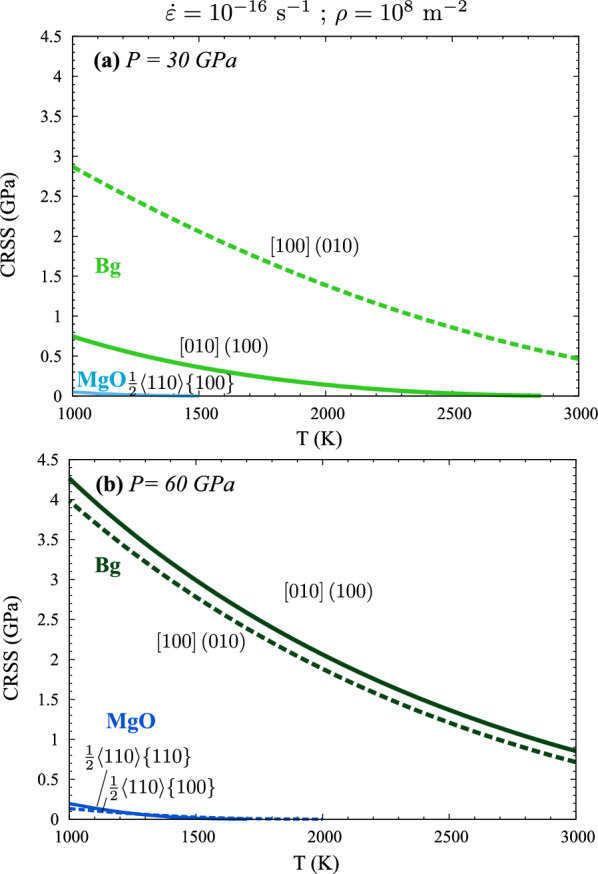
Influence of strain rate (here taken as a $\dot{\varepsilon }={10}^{-16} {s}^{-1}$) on lattice friction in MgSiO_3_ bridgmanite at two pressures: **a** 30 GPa, **b** 60 GPa (Kraych et al. 2016b). Reducing the strain rate to a value consistent with mantle convection effectively lowers lattice friction relative to Fig. 13. This effect is particularly significant for one of the slip systems at 30 GPa, but the stresses remain prohibitive for the other. At 60 GPa, lattice friction is incompatible with dislocation glide in MgSiO_3_ bridgmanite under lower-mantle conditions, despite the high temperatures

Lower strain-rates, such as those associated with mantle convection, allow more time for kink-pairs to nucleate under lower stress. Therefore, it is possible to take this effect into account when calculating the CRSS as a function of temperature for mantle strain-rates, eliminating the need for extrapolations. Figure 14 shows some results at P = 30 and 60 GPa, $\dot{\varepsilon }$=10^–16^ s^−1^ and a dislocation density ρ=10^8^ m^−2^. The choice of a lower dislocation density corresponds to the need to ensure a consistent mechanical balance with respect to mantle stresses. Indeed, Fig. 14a shows a significant decrease in the level of lattice friction compared to Fig. 13, especially for the [010](100) system. However, the general deformation of the crystal would require stress levels much higher than those expected in the mantle. This effect is even more pronounced at 60 GPa (Fig. 14b), corresponding to a depth of just 1500 km. Figure 12 illustrates what might occur at greater depths. Therefore, it appears that the lattice friction of bridgmanite is incompatible with the possibility of gliding of dislocations at mantle stress levels, even when considering the effects of temperature and strain rate.

Nevertheless, analysis of the core structures of dislocations suggests other possibilities, particularly the cores of edge dislocations, which exhibit a tendency, favored by pressure, to dissociate in the climb plane. In this case, a model of steady plastic flow by pure climb of dislocations (Nabarro 1967) is quite likely. This is what has been observed in MgAl_2_O_4_ spinel (Duclos et al. 1978), in Y_2_O_3_ (Gaboriaud 1981) and in SrTiO_3_ (Sigle et al. 2006). We therefore explored this avenue for MgSiO_3_ bridgmanite. To this end, Boioli et al. (2017) developed a modified 2.5D DD model to address pure climb, in which two orthogonal systems move by climb by exchanging vacancies. This numerical model yields results similar to those obtained with Nabarro’s (1967) analytical expression. Applied to bridgmanite under uppermost lower mantle conditions, it leads to creep rates that are fully compatible with those required for the mantle, even more efficient than those produced by diffusion creep once the grain size exceeds 0.1 mm (Boioli et al. 2017). Reali et al. (2019a) extended this approach based on Nabarro’s analytical model (1967) to the entire lower mantle along a geotherm. Within the limits of uncertainty regarding diffusion coefficients, which we will address below, the rheology of bridgmanite, as described by the pure climb creep model, appears to predict viscosities that are perfectly consistent with published viscosity profiles as a function of depth.

### The tale of wadsleyite and ringwoodite

We would now like to deviate slightly from the scope of this article on the lower mantle to recall the results obtained using the same approach on two minerals from the transition zone: wadsleyite and ringwoodite.

Following the procedure already described, the dislocations and slip systems properties of wadsleyite and ringwoodite have been modelled using the semi-continuum PNG model. GSF energies were computed at pressures corresponding to the transition zone conditions using either ab initio methods or empirical potentials. For wadsleyite, the easiest slip systems are $1/2\langle 111\rangle \left\{101\right\}$ and $\left[100\right]\left(010\right)$ (Ritterbex et al. 2016). For ringwoodite, the easiest slip systems are $1/2\langle 110\rangle \left\{110\right\}$ and $1/2\langle 110\rangle \left\{111\right\}$ (Ritterbex et al. 2015). In both cases, the dislocations exhibit core spreading, which leads to the formation of individual partial dislocations. Modelling the nucleation of kink-pairs—necessary for modelling thermal activation—required the elastic interaction model to be further developed mathematically. The validity of these additional developments was demonstrated through their prior application to the mechanical properties of SrTiO_3_ perovskite (Ritterbex et al. 2018).

**Fig. 15:**
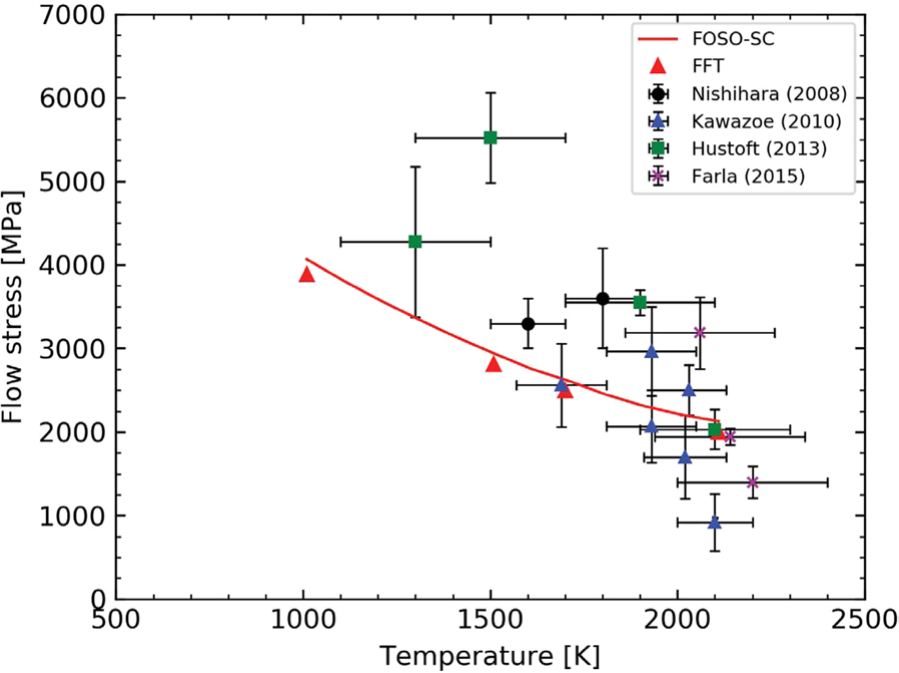
Macroscopic mechanical response of wadsleyite polycrystals deformed at 10^–5^ s^−1^ for various temperatures. Predictions of FOSO-Self-Consistent and FFT polycrystal models including dislocation mobility based on the GSF model are compared to experimental data from the literature. Modified after Castelnau et al. (2020). Note the very high level of stress

As with MgO and bridgmanite, it was possible to determine the evolution of CRSS with temperature at different strain-rates. Calculations at 10⁻5 s⁻1 enabled the numerical models to be validated by comparison with experimental data (Ritterbex et al. 2015, 2016). In the case of wadsleyite, additional effort was made to model the behavior of a polycrystal based on data determined at lower scales by Ritterbex et al. (2016). These data have been implemented in two grain-polycrystal scale transition models: a mean-field model (the Fully Optimized Second-Order Viscoplastic Self-Consistent scheme), which allows the effective viscosity of polycrystalline aggregates to be evaluated rapidly; and a full-field method based on fast Fourier transform (FFT), which allows stress and strain-rate localization in typical microstructures to be investigated (Castelnau et al. 2020). The agreement with experimental results is excellent (Fig. 15). With the MgO polycrystalline plasticity model of Amodeo et al. (2016), Castelnau et al. (2020) represents, at the time of writing, the most accomplished achievement in multiscale modelling of the plasticity of a mantle mineral.

CRSS calculations were also performed for wadsleyite and ringwoodite at a natural strain rate (Ritterbex et al. 2015, 2016). As with bridgmanite, although to a lesser extent, stress levels were found that preclude dislocation glide as a viable deformation mechanism in the transition zone. Applying the Nabarro (1967) model shows that pure climb creep is a credible alternative for deforming these phases in the mantle (Ritterbex et al. 2020). Moreover, it should be noted that in a layer where phase transitions occur, leading to a reduction in grain size at least locally, Nabarro–Herring-type diffusion creep remains a possibility.

### The chronicle of post-perovskite

MgSiO_3_ post-perovskite (ppv) is a mineral whose highly unusual crystallographic structure, exhibiting very strong anisotropy, poses particular problems for modeling. GSF calculations showed very early on (Carrez et al. 2007b; Goryaeva et al. 2015a) that this structural anisotropy had a significant impact on defects and mechanical properties. Unsurprisingly, the plane (010) of the layers appears to be the easy glide plane. The first results of dislocation calculations based on the PN (Carrez et al. 2007b) and PNG (Metsue et al. 2009) models showed, as expected, that the (010) plane of the sheets appears to be the easy-sliding plane. However, subsequent atomistic calculations showed that a more detailed description was required. While the spread of screw dislocation cores is a good indicator of the slip plane in other minerals, in ppv it can be seen that the [100] screws spread out in the {011} planes but slip in a zigzag pattern in the (010) plane (Goryaeva et al. 2015b). However, the [100](010) slip system is by far the easiest. Modeling its thermal activation using the line tension model shows that lattice friction vanishes at 500 K (Goryaeva et al. 2016). The ppv thus deforms very easily along [100](010) despite a pressure of 120 GPa. It is proposed that this very easy glide is not only the cause of good ductility, but that it must also contribute to the attenuation of seismic waves (Goryaeva et al. 2016). Second is the slip system [001](010), which confirms the ease of shearing of this plane (Goryaeva et al. 2017). However, this is not the complete picture, as a crystalline aggregate must accommodate strain in all three spatial directions to maintain compatibility. Carrez et al. (2017) showed that [010] dislocations are not stable in MgSiO_3_ ppv, leading to partial dislocations that may be linked to {110} mechanical twinning. This is potentially a major factor to consider in the development of CPOs and contributes to the plasticity of ppv outside the plane (010) without, however, solving the problem. What other mechanisms contribute to the three-dimensional deformation of ppv? Are they diffusive? Further studies or observations of experimental results are needed to make progress on this mineral.

## Discussion

### Which is weaker in the lower mantle—ferropericlase or bridgmanite?

Let’s start by pointing out that, in the field of rheology, the vocabulary can be confusing. For instance, it is meaningless to characterize a material simply by its *strength* as if it were an intrinsic property such as density. Strength is merely a behavior, a response to a given stress under specific physical and chemical conditions. Similarly, the words ‘strong’ or ‘weak’ implicitly refer to the ability to resist mechanical loading, and therefore ultimately to the strength of the bonds. This response could be evaluated using ideal shear or tensile stress calculations (see for instance Gouriet et al. 2019). In such a situation, we would naturally expect the oxide to be weaker than the silicates. However, things are more complicated, particularly in the case of time-dependent deformations, which activate various microscopic mechanisms. Mechanical behavior is then more accurately assessed by the deformation rate in response to a constant stress, the value of which can be decisive for behavior. This is why, in Cordier et al. (2023), we titled the paper ‘Periclase deforms more slowly than bridgmanite under mantle conditions’ rather than ‘Periclase is weaker than bridgmanite…’.

Under laboratory conditions, it is undisputed that periclase is weaker than bridgmanite (Girard et al. 2016). The same conclusion is reached (see, for example, Fig. 6 in Karato et al. 2025) when any attempt is made to extrapolate laboratory data to natural deformation rates using a creep law and a fixed set of parameters (activation energy *E*, stress exponent *n*, prefactor). This is because this approach implicitly assumes continuity of mechanisms (see Sect. 3.3). It is not easy to identify unambiguously the mechanisms involved (as there are often several). The parameters *n* and *E* are often used as indicators. However, as these parameters are often only apparent, they are difficult to interpret and cannot generally be linked unequivocally to a mechanism. Detailed analysis of microstructures at different scales remains the most effective method of identifying the mechanisms at work. While scanning electron microscopy (SEM) and electron backscatter diffraction (EBSD) are now widely used thanks to major advances in automated orientation mapping, it is unfortunate that detailed TEM investigations of defect microstructures remain underused, despite substantial progress in TEM methods for analyzing microstructures and defects.

Our models fully confirm the experimental results. Under conditions where the strain rate is imposed and the stress adjusts in response, the plasticity threshold will be reached much earlier in MgO (or ferropericlase) than in bridgmanite. This is shown in Fig. 13, where the CRSSs of the two phases are represented. And we agree that, under the same experimental conditions of imposed strain rate, even if it is much slower (Fig. 14), the activation of the same mechanisms (“glide”) would lead to the same conclusion.

Still, the boundary conditions in the mantle are different. Minerals must respond, at high temperatures, to low differential stresses resulting from buoyancy variations resulting from gravity acting on density differences. Depending on the activated mechanisms and their kinetics, the minerals respond with a given strain rate. In the case of bridgmanite, edge dislocations climb due to dislocation glide being inhibited by lattice friction and climb dissociation of the cores. In contrast, dislocations in ferropericlase can glide reasonably well; however, the deformation rate is limited by recovery (*r* factor of the Bailey-Orowan equation, see Sect.  3.1.2.2.2), which takes a considerable amount of time due to the slow kinetics of oxygen diffusion. Ultimately, bridgmanite deforms, but ferropericlase does not. Thus, the rheology of the lower mantle is controlled by bridgmanite. Since bridgmanite is simultaneously the most deformable phase and the volumetrically dominant one, phase morphology ceases to be a key parameter, in contrast to models where ferropericlase is assumed to be the deformable phase (Thielmann et al. 2020).

### How important is grain size, really?

Research on mantle rheology has long been polarized between diffusion- and dislocation-creep models. Because the former, assimilated to Nabarro-Herring creep, is grain-size dependent and the latter is not, grain size has been treated as a primary controlling parameter in geodynamical modeling (*e.g.* Solomatov 2001, Hall and Parmentier 2003, Solomatov and Reese 2008, Glišović et al. 2015, Dannberg et al. 2017, 2025, Paul et al. 2024). The key assumption underlying this class of models is that, in steady-state dislocation creep, grain size is in dynamic equilibrium between grain growth and grain-size reduction by dynamic recrystallization. Building on this framework, Ricard and Bercovici (2009) and Rozel et al. (2011) refined the treatment of aggregate evolution by modeling the dynamics of a grain-size distribution in a general and thermodynamically consistent way. The algebraic convenience of expressing rheology in terms of grain size has made this parameter a popular choice for numerical modeling. However, the flexibility of such models has sometimes led to grain size being allowed to vary over ranges that are physically unrealistic and incompatible with the underlying deformation mechanisms. It is therefore worth examining the physical basis of the assumptions behind these formulations.

#### Grain growth

While grain size is a standard parameter for rocks that can be characterized in the laboratory or on an outcrop, the same cannot be said for mantle rocks that cannot be observed directly. How can we infer grain size in the convecting mantle? The experimental way to access this parameter is to anneal fine-grained aggregates (very fine by necessity in the case of high-pressure experiments) at P, T and measure the evolution of grain size over time. This evolution can usually be described (Burke and Turnbull 1952) by:

16 $${d}_{f}^{n}-{d}_{i}^{n}=k.t$$

with ${d}_{f}$ and ${d}_{i}$, the final and initial grain radii, n the grain size exponent, *t* the experimental duration, and *k* is a grain growth rate. Grain growth is a thermally activated process, thus k usually is supposed to show Arrhenius type behavior:

17 $$k={k}_{0}\mathrm{exp}\left(-\frac{{E}_{a}}{RT}\right)$$

Here ${k}_{0}$ is a material-dependent pre-exponential factor and ${E}_{a}$ is the empirical activation energy for grain growth. These laws of evolution are subsequently extrapolated to geological timescales, often extending over hundreds of millions of years (Fei et al. 2023). Significant theoretical efforts were devoted to establishing a basis for Eq. 16 from the outset. The original model of Burke and Turnbull (1952) is based on capillarity where the driving force of grain growth is surface tension. Boundary migration occurs in response to a pressure due to surface curvature. The boundary tends to migrate toward its center of curvature reducing the area of the boundary and hence the surface energy. This model leads to n=2 although a wide range of values are effectively found (Burke and Turnbull 1952).

However, several recent findings have called this model into question. We will mainly mention studies focusing on SrTiO_3_, as it is more crystallographically similar to the minerals of interest here. Bäurer et al. (2009) and Rheinheimer and Hoffmann (2015) showed that the growth dynamics in SrTiO_3_ does not follow the classical Arrhenius-type temperature dependence. The existence of different growth regimes is interpreted as the results of different grain boundary types, a fast low temperature type and a slow high temperature type (Rheinheimer and Hoffmann 2015). In 2021, Bhattacharya et al*.* characterized 52,000 grain boundaries in a nickel polycrystal annealed at 1073 K. They found no correlation between grain boundary velocity and curvature. A similar observation was made in 2023 by Muralikrishnan et al*.*, but this time on SrTiO_3_. While the reduction of grain-boundary energy (capillarity), as in classical models, remains the ultimate driving force for grain growth, the assumption that grain boundaries migrate toward their centers of curvature at a speed proportional to the local curvature—like in soap froths—can no longer be maintained (Rohrer et al. 2023). A proper description of grain-growth kinetics would require accounting for the nature of grain boundaries (complexions), their defect structure (disconnections), and their elastic coupling in the grain-boundary motion equation (Qiu et al. 2025).

#### Grain size reduction

In this discussion, we will focus on models that consider an equilibrium grain size resulting from a steady-state deformation regime (Rozel et al. 2011). Therefore, we will only consider dynamic recrystallisation as a mechanism for grain size reduction, and not phase transformations. In this case, the driving force is the elastic energy stored in the deformed grains, which contain significant dislocation densities. This phenomenon is commonly observed in experiments and in the lithosphere. However, it should be noted that this driving force is usually related to the stress applied during deformation (Guillopé and Poirier 1979). The stored strain elastic energy is of the order of:

18 $$\frac{1}{2}\rho \mu {b}^{2}$$

ρ is the dislocation density, μ is the shear modulus and *b* is the modulus of the Burgers vector. In order for the mineral to be in mechanical equilibrium, the back stress from stored dislocations must balance the deviatoric stress. For mantle stresses estimated to be between 1 and 10 MPa, the dislocation density should be between 10^8^ and 10^10^ m^−2^. The grain boundary energy is:

19 $$\frac{2{\gamma}_{gb}}{R}$$

**Fig. 16:**
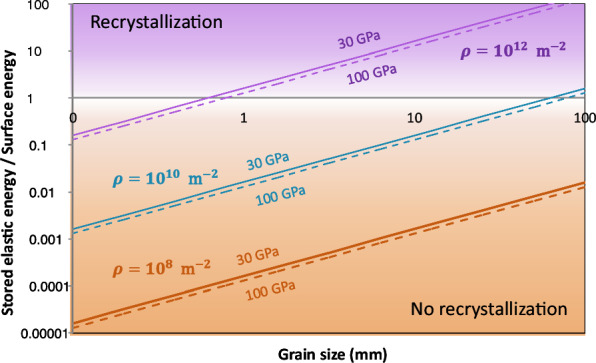
Driving force for dynamic recrystallization estimated from the ratio of the stored elastic energy (dislocations, Eq. 18) to grain boundary surface energy (Eq. 19). Data from Gouriet et al. (2014)

${\gamma}_{gb}$ is the grain boundary surface energy, *R* is the grain radius. For recrystallization to occur, the stored strain energy should exceed the grain boundary surface energy. Figure 16 shows that, under mantle conditions (*i.e.* creep under low stress corresponding to low dislocation densities), the conditions are not favorable to promote dynamic recrystallisation. The hypothesis of an equilibrium grain size does not seem acceptable, and further research is needed to explore this issue.

### Should Nabarro (pure climb) creep and diffusion creep really be opposed?

It is important to remember that nature is unaware of the discontinuous nature of our conceptual approaches. Let us consider deformation mechanisms in which transport of matter is significant or even dominant, as opposed to transport of shear (dislocation glide). In order to produce plastic deformation, transport of matter must extend beyond simple random walk of atoms resulting from thermal agitation and become oriented by the applied stress. In the Nabarro-Herring model, the difference in vacancy concentration in the vicinity of grain boundaries under compression or tension generates a gradient along which the vacancies flow. Depending on their state of loading, the different grain boundaries then act as sources or sinks of vacancies (Fig. 2a). The resulting flow of matter produces strain. Grain boundaries are not the only defects that can play this role. Surfaces can also do so, as can, more importantly in the context of our problem, dislocations. The different situations proposed in the literature are: individual dislocations (Nabarro (pure climb) creep, Nabarro 1967, Fig. 2c); sub-grain boundaries (subgrain creep, Weertman 1968, Fig. 2b); and grain boundaries (Nabarro-Herring creep, Herring 1950, Fig. 2a). The characteristic length *L*_*i*_ (Fig. 2), which is the distance between sources and sinks, controls the efficiency of the diffusive process. This characteristic length is determined by microstructural parameters such as dislocation density (Fig. 2c), cell size (Fig. 2b, this configuration is more common in metals) or grain size (Fig. 2a). This variety is important because, as discussed above, grain size is not as flexible a parameter in mantle rocks as it is in models.

The slowing down of diffusion under high pressure imposes rather stringent constraints on grain size. Even though these constraints remain to be fully quantified, their consequences can be described. Grain growth is limited, and the capacity of grain size to respond or adjust to changing conditions (stress, strain-rate, temperature) is intrinsically slow. Combined with the absence of a credible mechanism for significant grain-size reduction in the convecting mantle, this implies that grain size cannot be regarded as a sufficiently flexible degree of freedom to allow rocks to continuously adjust their viscosity, as commonly assumed in numerical models. By contrast, rocks possess much more adaptable microstructural degrees of freedom at the intragranular scale, such as dislocations and subgrain structures. These internal microstructures can evolve more flexibly and rapidly, allowing the rock to accommodate creep under mantle conditions. A continuous transition from Nabarro–Herring diffusion creep to Nabarro (pure climb) creep through evolution of dislocation density is therefore a plausible mechanism.

### How do our models compare with natural observations?

To assess the relevance of these models, it is essential to confront their predictions with natural observables. This step is important and necessary, but we must remain aware of the difficulties it presents. Accurately accounting for the rheology of the relevant phases under the P, T and $\dot{\varepsilon }$ conditions of the lower mantle remains an ambitious goal, whether through experimental approaches or modelling deformation mechanisms. Although the coarse-graining from minerals to aggregates or polyphase polycrystals has been addressed, this remains a work in progress. Therefore, at best, we are only able to account for the rheology of a very small representative volume compared to the scale of mantle convection. Other geodynamic modelling approaches are needed to enable us to compare our results with observations. However, some broad conclusions can be drawn; the first of these relates to seismic anisotropy.

The absence (or very low level) of seismic anisotropy throughout the lower mantle has been the main argument in favor of diffusion creep. However, Nabarro (pure climb) creep, which generates strain along ${\boldsymbol{b}}\otimes {\boldsymbol{b}}$ without rotation, also does not produce crystal preferred orientations and therefore does not generate seismic anisotropy. This mechanism is therefore consistent with the absence of seismic anisotropy signatures observed in the lower mantle (the D" layer will be discussed separately). Viscosity, as deduced from various observables such as glacial isostatic adjustment and the geoid, is another factor that our models must consider. The concept of a spherically symmetric Earth has been disregarded for a long time, and lateral variations in viscosity are clearly important. However, the viscosity of the mantle is frequently depicted as a radial profile that aims to account for the first-order pressure effect. Reali et al. (2019a) showed that a lower mantle whose rheology could be described by the Nabarro (pure climb) creep of bridgmanite is entirely consistent with the radial viscosity profiles reported in the literature. The uncertainties of the model, expressed through the current lack of knowledge about the concentration of vacancies, are even capable of describing radial or lateral variations. However, it seems to us that, in the absence of more precise constraints, such an approach is currently irrelevant.

One of the points that distinguishes Nabarro (pure climb) creep from diffusion creep is the non-linearity of rheology and the existence of a yield stress due to internal stress caused by the presence of dislocations. This last characteristic is one of the components that can account for the existence of fat plumes observed by seismic tomography, as shown by Davaille et al. (2018).

In addition to GIA and geoid observations, global observations of slab sinking provide a direct in situ constraint on lower-mantle rheology. Age-depth correlations of subducted slab remnants reveal a robust trend of sinking through the lower mantle at rates of approximately 1–1.5 cm/yr, largely independent of assumptions on mantle rheology. Numerical mantle-flow experiments incorporating a pure dislocation climb creep (PCC) rheology reproduce this observed sinking behavior for a narrow range of atomic self-diffusion coefficients. The confrontation between modeled and observed slab sinking curves therefore allows the self-diffusion coefficient—and hence the effective viscosity of the lower mantle—to be quantitatively bounded. In this framework, slab sinking velocity becomes a measurable observable against which the viability of the pure climb creep mechanism can be directly tested (Spakman et al. 2025).

Until now, we have sought to distinguish the discussion of the D" layer from that of the lower mantle as a whole. This is not because it is not part of the lower mantle, but because it differs from it in several ways. Firstly, as the hot thermal boundary layer of convection, it is the site of significant temperature, flow and composition gradients. The exact nature of structures such as LLSVPs or ULVZs is still subject to debate. Secondly, in the coldest areas, bridgmanite is likely to transform into post-perovskite. The properties of this special phase must therefore be considered in relation to the characteristics of the D" layer. We have shown that it is highly anisotropic from both a structural and a rheological point of view, with particularly easy dislocation slip in the (010) plane and twinning deformation (Goryaeva et al. 2015b, 2017; Carrez et al. 2017). Unlike the lower mantle as a whole, the D" layer exhibits significant seismic anisotropy. Even if factors other than CPOs are responsible for seismic anisotropy, a connection must be established. Furthermore, we have demonstrated that, in contrast to bridgmanite, [100] dislocations in post-perovskite are not subject to lattice friction, with the athermal plateau being reached at an earlier stage than in MgO. They can therefore be set in motion by very weak forces, such as those generated by the passage of a seismic wave. This type of inelastic behavior can lead to seismic attenuation (Goryaeva et al. 2016), which is consistent with reports of higher attenuation in the D″ layer by Anderson and Hart (1978) and Lawrence and Wysession (2006). We emphasize that the rheology of post-perovskite has not yet been fully described or modelled. Nevertheless, the existence of such easy slip in the basal plane suggests low viscosity, which probably has implications for flow in the D″ layer and may promote heat transfer from the core through the CMB (Buffett 2007; Nakagawa and Tackley 2011; Li et al. 2014). While definitive conclusions cannot be drawn, it is worth noting that the properties we predict for post-perovskite are consistent with certain observable phenomena in the D″ layer.

### Diffusion: the hidden driver of mantle rheology

All of our models lead to the same conclusion: the rheology of the lower mantle depends primarily on ionic diffusion, whether for bridgmanite or ferropericlase, although the mechanisms involved are different. Therefore, it is reasonable to conclude that the robustness of our models depends on the quality of the diffusion data. There are a number of difficulties in acquiring experimental diffusion data for mantle minerals, particularly for the slowest diffusing elements that control the creep rate. One issue is that the diffusion length scales are often very short, making it difficult to accurately measure the isotope concentration profiles that are typically used to determine self-diffusion coefficients. Another problem is the enhancement of diffusion by structural defects, which can dominate bulk transport for elements that diffuse slowly in the lattice. Both of these issues are amplified at high pressures, where it can be difficult to recover samples with surfaces suitable for high-precision depth profiling analyses, and where dislocations and other structural defects are likely to be produced during compression. These problems, and the need to extrapolate diffusion data to pressures and temperatures beyond the reach of laboratory experiments, make it necessary to rely to a large extent on theoretical calculations of elemental diffusivities.

For periclase, decades of experimental and theoretical work on MgO, reviewed by Van Orman and Crispin (2010), have produced a mature understanding of the mechanisms and rates of diffusion, and their temperature and pressure dependences. In both high-purity synthetic MgO and natural periclase, Mg diffusion is due to extrinsic cation vacancies produced to balance the charge of trivalent impurities, whereas O diffusion is due to intrinsic Mg-O vacancy pairs that are insensitive to impurity concentrations. Due to the large formation energy for Mg-O vacancy pairs, oxygen vacancies have very low concentrations, and diffusion of O is accordingly much slower than Mg. Diffusion data for O among different experimental studies show considerable dispersion, over several orders of magnitude, which may be due to pipe diffusion along dislocation cores in samples with a wide range of dislocation densities (Van Orman and Crispin 2010; Landeiro dos Reis et al. 2022). True intrinsic bulk diffusion of O does seem to have been measured in CVD-grown crystals with very low defect concentrations, at temperatures > 2000 K (Yang and Flynn 1994). These intrinsic O diffusion coefficients are in good agreement with theoretical calculations (Ita and Cohen 1997; Karki and Khanduja 2006; Ammann et al. 2010). Experimental measurements at high pressures (Van Orman et al. 2003) are complicated by the sensitivity of O diffusion to structural defects such as dislocations, which may be produced during compression when using solid pressure media. Oxygen diffusion coefficients obtained at pressures up to 25 GPa were clearly affected by such defects, whereas the Mg diffusion measurements in the same study were not, due to the higher (extrinsic) cation vacancy concentration. It would be very difficult if not impossible to perform experiments, at suitably high pressures, which are in the intrinsic O diffusion regime that is relevant to mantle creep. Hence, it is essential to rely on theoretical calculations, which predict a very strong pressure dependence for O diffusion in MgO and very slow diffusion under lower mantle conditions (Fig. 10). The extremely slow intrinsic diffusion of O could be enhanced along dislocations (Van Orman et al. 2003; Landeiro dos Reis et al. 2022) or grain boundaries (McKenzie et al. 1971; Hashimoto et al. 1972; Gordon 1973; Van Orman et al. 2003; Riet et al. 2021). However, the density of dislocations, and MgO grain or sub-grain boundaries, in the mantle are probably too low to have a significant effect in the mantle.

An important question is how the composition of periclase changes its oxygen diffusion and therefore creep properties. While impurities such as Fe^3+^ and Al^3+^ have negligible effects on O diffusion (Yang and Flynn 1994; Ando et al. 1983), solid solution of FeO, on the other hand, is expected to have a significant effect, because it results in weaker bonds, depresses the melting temperature, and should lower the formation energy for Schottky defects. Reali et al. (2019b) showed that for materials with the rock salt structure, including MgO and FeO, the intrinsic anion diffusion coefficient is remarkably similar at the same fraction of the melting temperature *T*_*m*_, and used this homologous temperature scaling to predict the oxygen diffusion coefficients for Fe-rich (Mg,Fe)O, which have not been determined experimentally. This approach has inherent uncertainties, however. MgO and FeO are mutually soluble across their whole range of composition, but there is a wide gap between their solidus and liquidus curves, and no clear guidance from experiment or theory on whether the solidus or liquidus temperature—or a temperature intermediate between them—is more appropriate for scaling the diffusion coefficients. Furthermore, experiments show anomalous behavior of the solidus and liquidus temperatures with maxima at ~ 40 GPa (Deng and Lee 2017). Reali et al. (2019b) assumed for simplicity that the “effective” melting temperature of the solid solution at the pressure of interest was the linear interpolation between the melting temperatures of MgO and FeO, but experiments and/or theoretical calculations are needed to directly address the diffusivity of oxygen in (Mg,Fe)O solid solutions.

Bridgmanite is a more difficult object for diffusion studies because it is stable only above ~ 22 GPa and has a more complex chemistry than MgO. Accordingly, our understanding of the mechanisms and rates of diffusion is not as well developed as it is for periclase. Bridgmanite is composed primarily of MgSiO_3_ but with substantial Al^3+^ and Fe^3+^ in solid solution. These trivalent cations could be distributed equally between the two cation sites, normally occupied by Mg^2+^ and Si^4+^, respectively, to maintain charge balance. However, they tend to preferentially occupy the octahedral Si site, where they are charge-balanced by oxygen vacancies (Dou et al. 2025; Fei et al. 2021). Oxygen vacancies are therefore the primary mobile point defects, at least at moderate pressures, and oxygen is the fastest diffusing species (Dobson et al. 2008; Dou et al. 2025). Therefore, cation diffusion on either the Mg or Si sub-lattice is expected to be the rate − limiting step for dislocation climb in bridgmanite. Experimentally, self − diffusion coefficients for Mg and Si have only been measured at pressures relevant to the top of the lower mantle (~ 25 GPa) based on multi − anvil experiments combined with secondary ion mass spectrometry depth profiling (Yamazaki et al. 2000; Dobson et al. 2008; Xu et al. 2011). In all cases, the diffusion length scale was very short (less than 200 nm), which is near the resolution limit for SIMS depth profiling of high-pressure samples that undergo surface roughening during the diffusion anneal, and the profiles were interpreted to reflect the convolution of both lattice and grain boundary diffusion. Data are also available for Fe–Mg interdiffusion, based on TEM measurements of the experimental diffusion profiles (Holzapfel et al. 2005). The experiments indicate that Si, Mg and Mg–Fe all appear to have comparable lattice diffusivities. In contrast, first-principles calculations predict a much lower concentration of Si vacancies than Mg vacancies, suggesting that intrinsic diffusion of Si should be much slower than Mg (Dou et al. 2025). Based on first-principles and atomistic simulations (Ammann et al. 2009, 2010; Dou et al. 2025), intrinsic Si diffusion in bridgmanite should be much slower than has been measured in experiments, and much too slow to explain the viscosity of the lower mantle (Cordier et al. 2023). This suggests that some kind of extrinsic defect controls Si diffusion under both experimental and natural conditions, despite the predominance of extrinsic oxygen vacancies, which suppress extrinsic cation vacancies. What these extrinsic defects might be, their concentrations, and the corresponding Si diffusivities in the lower mantle remain open questions. An important step to addressing these questions would be a comprehensive model of the point defect populations in (Mg,Fe,Al)(Si,Fe,Al)O_3_ bridgmanite, similar to the approach used by Hirsch and Shankland (1991) for (Mg,Fe)SiO_3_ bridgmanite, but with improved constraints from first-principles calculations of the thermodynamic parameters for the relevant point defect reactions.

## Conclusion

Together, the developments reviewed here show that mineral-physics-based multiscale modelling has evolved from a collection of isolated approaches into a coherent framework capable of linking atomic-scale defect processes to macroscopic mantle rheology under extreme conditions. While significant uncertainties remain, notably in diffusion data and defect chemistry, the methodology now provides a physically grounded pathway to bridge the vast gap between laboratory timescales and planetary deformation.

We emphasize that, as long as one adheres strictly to available data, the outcomes of numerical and experimental approaches are remarkably consistent. Divergences emerge primarily when these data are extrapolated beyond their domain of validity, or when they are used to interpret phenomena occurring at spatial or temporal scales far removed from those directly constrained by observation. In this sense, apparent discrepancies between models and experiments are often artificial, and should be viewed as an invitation to place greater weight on robust data rather than on unconstrained extrapolation.

Continued progress in experimental, theoretical, and computational approaches now places the community in a position to transform mantle rheology from a discipline largely dominated by extrapolation into one increasingly constrained by mechanism-based, physically grounded predictions.

## Data Availability

‘Not applicable’.

## Footnotes

1 Hall–Petch law describes the increase of the yield strength (${\sigma}_{y}$) of a polycrystal as the grain size (*d*) decreases, and it is commonly expressed as ${\sigma}_{y}={\sigma}_{0}+k{d}^{-1/2}$

2 this is the name given to dislocations that intersect the glide plane.

3 Forest dislocations are dislocations lying on different slip systems that intersect mobile dislocations and impede their motion.
